# Unique and Repeated Stwintrons (Spliceosomal Twin Introns) in the Hypoxylaceae

**DOI:** 10.3390/jof8040397

**Published:** 2022-04-13

**Authors:** Erzsébet Fekete, Fruzsina Pénzes, Norbert Ág, Viktória Ág-Rácz, Erzsébet Sándor, Claudio Scazzocchio, Michel Flipphi, Levente Karaffa

**Affiliations:** 1 Department of Biochemical Engineering, Faculty of Science, University of Debrecen, H-4032 Debrecen, Hungary; penzesgirl03@gmail.com (F.P.); agnorbi@gmail.com (N.Á.); rcz.viktoria@gmail.com (V.Á.-R.); drir.michelflipphi@gmail.com (M.F.); levente.karaffa@science.unideb.hu (L.K.); 2 Juhász-Nagy Pál Doctoral School of Biology and Environmental Sciences, University of Debrecen, H-4032 Debrecen, Hungary; 3 Institute of Food Science, Faculty of Agricultural and Food Science and Environmental Management, University of Debrecen, H-4032 Debrecen, Hungary; karaffa@agr.unideb.hu; 4 Section of Microbiology, Department of Infectious Diseases, Imperial College London, London SW7 2AZ, UK; c.scazzocchio@imperial.ac.uk; 5 Institute for Integrative Biology of the Cell (I2BC), Université Paris-Saclay, CEA and CNRS Unité Mixte de Recherche UMR 9198, 91190 Gif-sur-Yvette, France; 6 Institute of Metagenomics, University of Debrecen, H-4032 Debrecen, Hungary

**Keywords:** spliceosomal introns, splicing, stwintrons, comparative genomics, stwintron dissemination, stwintron propagation, internal symmetry, single-stranded RNA secondary structure prediction, *Hypoxylon* sp. CO27-5, Hypoxylaceae

## Abstract

Introns are usually non-coding sequences interrupting open reading frames in pre-mRNAs [D1,2]. Stwintrons are nested spliceosomal introns, where an internal intron splits a second donor sequence into two consecutive splicing reactions leading to mature mRNA. In *Hypoxylon* sp. CO27-5, 36 highly sequence-similar [D1,2] stwintrons are extant (sister stwintrons). An additional 81 [D1,2] sequence-unrelated stwintrons are described here. Most of them are located at conserved gene positions rooted deep in the Hypoxylaceae. Absence of exonic sequence bias at the exon–stwintron junctions and a very similar phase distribution were noted for both groups. The presence of an underlying sequence symmetry in all 117 stwintrons was striking. This symmetry, more pronounced near the termini of most of the full-length sister stwintrons, may lead to a secondary structure that brings into close proximity the most distal splice sites, the donor of the internal and the acceptor of the external intron. The *Hypoxylon* stwintrons were overwhelmingly excised by consecutive splicing reactions precisely removing the whole intervening sequence, whereas one excision involving the distal splice sites led to a frameshift. Alternative (mis)splicing took place for both sister and uniquely occurring stwintrons. The extraordinary symmetry of the sister stwintrons thus seems dispensable for the infrequent, direct utilisation of the distal splice sites.

## 1. Introduction

Originally, introns were defined as non-coding sequences interspersed with exons in primary transcripts (pre-mRNA), which when accurately excised generated the open reading frame (ORF) in mature mRNAs. Spliceosomal introns (U2 introns) are a hallmark of Eukaryota and widespread in nuclear transcriptomes, with average densities of three to four introns per gene in Pezizomycotina (e.g., [1]). Group-I and Group-II introns predominantly exist in Prokaryota and in organelle genomes in eukarya (mitochondria, plastids), and they catalyse their own excision, sometimes with the aid of accessory proteins (for reviews, see [2,3]). These ribozymes fold into complex secondary and tertiary structures that are indispensable for self-splicing as well as for the process of retro-insertion or retro-transposition, the mechanism by which Group-II introns proliferate [4,5,6,7]. By contrast, precise and expeditious excision of spliceosomal introns requires a huge ribonucleoprotein (RNP) complex, called the U2 spliceosome, and crucially involve the intron’s terminal 5′-donor and 3′-acceptor Gs at the respective splice sites and the lariat branch point (BP) adenosine as its prime cis-acting elements (for reviews, see [8,9]). The five small nuclear RNAs (snRNAs: U1, U2, U4, U5 and U6) are indispensable for recognising intron splice sites and the branch point to assemble the spliceosome and to catalyse the two phospho-transesterification reactions necessary to remove the intron and ligate the bordering exons [10,11,12]. Intron RNA is usually rapidly turned over after re-linearisation of the lariat RNA released. Nevertheless, some intron RNAs are more resilient and carry out crucial functions after their excision from pre-mRNAs (for reviews, see [13,14,15]).

**Figure 1:**
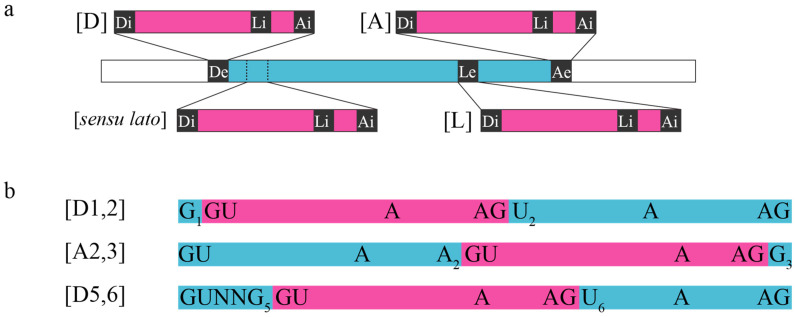
Stwintrons are complex intervening sequences of nested canonical U2 introns excised by consecutive standard splicing reactions. (**a**) Classes of stwintrons. Stwintrons are the spliceosomal analogues of the originally described group II/III twintrons (twin introns) in plastid genomes of *Euglena gracilis*. Stwintrons can occur in four classes, depending on the location where the internal U2 intron interrupts the continuity of the external U2 intron: [D], in the latter’s 5′-donor; [L], in its canonical lariat branch point (BP) element; [A], in its 3′-acceptor; [sensu lato], canonical intron within canonical intron, where the internal intron does not disrupt the [D], [L] or [A] elements of the external intron. The internal intron is shown in magenta, the external intron in turquoise. (**b**) Structure of the [D1,2], [A2,3] and [D5,6] types of stwintrons. The schemes show the exact site of the continuous internal intron located within the split external intron sequence: the internal U2 intron must be excised from the pre-mRNA before the external U2 intron can be spliced out and the bordering exons are properly fused [18]. When a G is the first exonic nt downstream of the most 3′-splice site of a [D1,2] (5′-HAG|G), the complex intervening sequence can be alternatively removed as an [A2,3] stwintron located one nt to 3′, the two alternative splicing paths yielding the same continuous exonic sequences [23,24].

Complex intervening sequences consist of nested or recursive canonical U2 introns that require consecutive splicing reactions to be properly excised (for reviews, see [16,17]). Stwintrons (spliceosomal twin introns) [18] are the spliceosomal analogues of the original Group-II/III twin introns (twintrons) in the plastid genome of *Euglena gracilis* ([19]; n.b., Group-III introns are abbreviated versions of Group-II introns), where an internal intron disrupts a sequence element necessary for the excision of an external intron. Stwintrons can be classified into four classes (Figure 1a). Usually, the internal U2 intron disrupts the continuity of either the consensus 5′-donor [D], branch point (BP) sequence element [L] or 3′-acceptor [A] of the external U2 intron. In addition, we have described functional stwintrons where the internal U2 intron is integrated into the external intron next to, rather than within, the latter’s consensus 5′-donor [20]. In a [D1,2] stwintron (Figure 1b), the internal intron is located in the 5′-donor sequence of the external intron between the first and the second nucleotides (nts) (5′-G_1_|U_2_RWGY) [18,21]. If the internal splice sites of a [D1,2] are masked by local double-stranded RNA or an RNA-binding protein, the intervening sequence is not completely removed by one standard splicing reaction, and the first G of the [D1,2] stwintron becomes exonic, leading to a frameshift (+1). In a [D5,6] stwintron (Figure 1b), the internal intron is located in the external intron’s donor sequence between the fifth and the sixth nt (5′-GURWG_5_|Y_6_) [22]. In a [A2,3] stwintron, however, the internal intron is located in the 3′-acceptor sequence of the external intron between its penultimate and ultimate nts (5′-HA_2_|G_3_) [23,24]. The geometry of the constituent introns of overlapping [D1,2] and [A2,3] stwintrons—two genuine U2 introns in the genome sequence overlapping at their terminal G (5′-AGT)—is such that a [D1,2] stwintron can alternatively be removed as a [A2,3] stwintron of virtually the same sequence, when a G is present directly downstream of the most 3′ acceptor sequence.

Previously, we identified 23 [D1,2] sister stwintrons of high-sequence similarity over the complete width of the intervening sequence and another 13, named sheared sister stwintrons, with high similarity restricted to a section thereof. Most of these 36 sequence-related stwintrons are exclusive to the narrow taxon of *Hypoxylon* sp. strains CO27-5 and EC38 [25].

We also found 12 canonical introns in that taxon derived from an ancestor sister stwintron, ten of which exhibited very strong internal symmetry, as there is no spacer between the terminal inverted repeats so typical for the sister stwintrons. During the analysis of two of the genes carrying such “multiple occurring” (MO) intervening sequences, we found two [D1,2] stwintrons, the sequences of which showed no obvious similarity with those of the sister stwintrons. In the current work, we employed a simple motif search tool to identify additional “uniquely occurring” (UO) [D1,2] stwintrons while screening *Hypoxylon* sp. CO27-5 DNA sequence contigs. The existence of 117 [D1,2] stwintrons was established and two-thirds of these were stwintrons that had been not identified earlier by sequence similarity. The overwhelming majority of the newly identified UO [D1,2] stwintrons are located at gene positions that are also occupied by “orthologue” stwintrons in the orthologue genes in 16 other taxa of Hypoxylaceae, resulting in the identification of more than 1500 [D1,2] stwintrons.

## 2. Materials and Methods

### 2.1. Motif Search Tool for Identifying Stwintrons in Whole Genome Sequence Contigs

**Figure 2:**
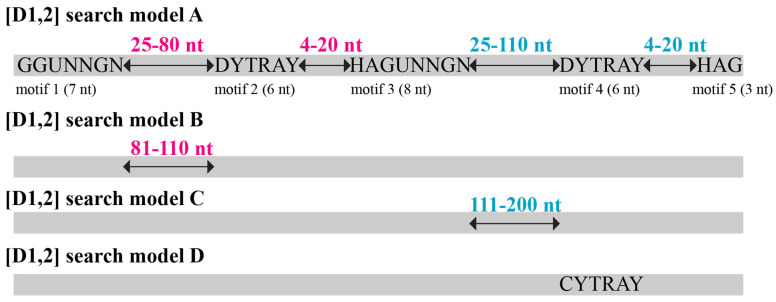
Graphic scheme of the stwintron search models employed to identify candidate [D1,2] stwintrons occurring in DNA contigs. Five degenerated sequence motifs (5′ to 3′ numbered as motifs 1–5) consisted of the consensus 5′-donor, BP element and 3′-acceptor sequences from the constituent U2 introns and comprised two hybrid motifs that included nts from both internal and external introns (motifs 1 and 3, respectively). These hybrid motifs were specific to the type of stwintron (i.e., [D1,2], [D2,3], [D3,4], [D4,5] or [D5,6] for [D] class stwintrons). The distance ranges between consecutively degenerated sequence motifs are defined as detailed in Section 2 and Section 3. The set distance ranges within the internal intron are printed in red lettering and those within the external intron in blue lettering. The initial search was carried out with [D1,2] Search Model A, which identified 90 *Hypoxylon* sp. CO27-5 [D1,2] stwintrons. Below the graphic scheme of Search Model A, abbreviated schemes for three further search models ([D1,2] Search Models B, C and D, respectively) show only the variables that were changed. These three secondary screens identified 22 additional [D1,2] stwintrons missed by Search Model A.

We analysed the *Hypoxylon* sp. CO27-5 genome sequences [26] with a sequence motif search algorithm that allows for the screening of genome sequence contigs for putative stwintrons. This motif search is far less reliant on sequence similarity than standard BLASTN screens. The principles of this tool were described in Fekete and co-workers [24] and involve degenerated motifs for filamentous fungal U2 intron 5′-donors (6 nt), lariat BP sequences (6 nt) and associated 3′-acceptors (3 nt) (cf., [27]), including two hybrid sequence motifs that consisted of nts of the external as well as the internal intron. These hybrid motifs are specific for each one of the possible types of stwintrons. For [D1,2] stwintrons (Figure 2), the 5′-hybrid motif (motif 1; 7 nt) necessarily starts with a 5′-G (i.e., the G_1_ of the split 5′-donor of the external intron at the 5′ of the stwintron) followed by the full donor sequence of the internal intron, while the 3′ of the BP motif of the internal intron (motif 2), the second hybrid motif (motif 3; 8 nt), consisted of the 3′-acceptor of the internal intron (5′-HAG) plus the remainder of the split 5′-donor sequence of the external intron 5′-U_2_RWGY (n.b., the 5′-GURWGY canonical donor as defined in [27]). The 3′-hybrid motif at the fusion of the constituent internal and external introns precedes the BP motif of the external intron (motif 4) and the associated 3′-acceptor at the 3′ end of the stwintron (motif 5). Figure 2 shows a graphical scheme of the primary [D1,2] stwintron search model A. We included a wide variety of potential 5′-donor sequences of 5′-G_1_T_2_N_3_N_4_G_5_N_6_ strictly biased at positions 1, 2 and 5, but we only searched with 24 of the 64 possible donor sequences, namely, those that each individually represented >0.6% of genuine intron donors in a statistical analysis of hundreds of [D5,6] stwintrons across Pezizomycotina species (our unpublished results). The [D5.6] stwintron in the ubiquitous reticulon-like gene was present across almost all Pezizomycotina classes [22], and it is arguably the oldest stwintron described to date. The initial degenerated lariat branch point sequences and associated 3′-acceptors, 5′-D_1_YTRAY and 5′-H_1_AG, respectively, were also supported by the reticulon-like gene [D5,6] analysis. To keep the search results manageable, we thus precluded the use of less-canonical intron sequence elements, such as the 5′-GC_2_ for donor, 5′-RCC_3_AAY for BP or 5′-G_1_AG for acceptor, which are rarely extant in filamentous fungal U2 introns.

Furthermore, we defined distance ranges between the five short [D1,2] stwintron sequence motifs at the 5′- and 3′-splice sites, and the conserved sequence elements, including the branch point As (adenosines) from our statistical analysis of the [D5,6] stwintrons in Pezizomycotina reticulon-like genes (see Figure 2, Model A). Distances between the donor and BP sequences were always longer than those between the BP and their associated acceptors and, in the large majority of cases, much longer. The minimum distance between BP and acceptor was fixed at 4 nt. The minimum length of a canonical U2 intron was set at 42 nt. The genome sequences of *Hypoxylon* sp. CO27-5 (master accession number: MDCL01) consist of 580 sequence contigs. The contigs were downloaded from the National Center for Biotechnology Information (NCBI) servers and screened with the stwintron sequence motif search model A using the DNA Pattern Find program from the Sequence Manipulation Suit [28]. The exact formula of the [D1,2] motif search model A is printed below, showing all 24 hexamer donor sequences considered, for both the internal intron and the external intron (underlined: the 5′-G of the [D1,2] stwintron is part of the split donor (5′-G_1_|T_2_) of its external intron). The numbers between accolades are the space ranges set between the five conserved sequence motifs in [D1,2] stwintrons.

G(GTAAGT|GTATGT|GTGAGT|GTACGT|GTAAGC|GTATGC|GTAGGT|GTAAGA|GTGCGT|GTACGC|GTGAGC|GTATGA|GTAAGG|GTTAGT|GTCAGT|GTGAGA|GTGTGT|GTTTGT|GTACGA|GTATGG|GTTCGT|GTGGGT|GTAGGC|GTACGG).{25,80}[AGT][CT]T[AG]A[CT].{4,20}[ACT]AGT(AAGT|ATGT|GAGT|ACGT|AAGC|ATGC|AGGT|AAGA|GCGT|ACGC|GAGC|ATGA|AAGG|TAGT|CAGT|GAGA|GTGT|TTGT|ACGA|ATGG|TCGT|GGGT|AGGC|ACGG).{25,110}[AGT][CT]T[AG]A[CT].{4,20}[ACT]AG

### 2.2. Screening Extant Sequence Read Archives (SRAs)

In *Hypoxylon* sp. CO27-5, most of the gene model predictions could be corroborated by RNA sequence reads (the six relevant SRA accessions listed in Table 1). We identified perfectly matching SRA reads (size: 100 nt) by BLASTN screening of the NCBI’s Sequence Read Archive for *Hypoxylon* sp. CO27-5 using 60 nt long query sequences containing the predicted exon/exon fusion site at its centre. To detect the stwintron splicing intermediate (splinter)—the RNA species that lacks the predicted internal intron of the [D1,2] stwintron but still contains its external intron—we used query sequences (60 nt) containing in their centre, the predicted fusion site of the upstream exon and the external intron of the stwintron with its functional donor sequence intact. SRAs that confirm putative stwintrons and their predicted splinter are listed in Supplementary Materials Table S1 (sheet CO27-5). Occasionally, we searched and found SRAs from *Hypoxylon* sp. EC38 (the six relevant SRA accessions listed in Table 1) that validated the stwintron (n.b., in almost all cases, present in both allied strains) and/or its typical splicing intermediate in the absence of covering reads from CO27-5. These corroborative EC38 SRA reads are marked in bold blue letters on a light grey background in Supplementary Materials Table S1 (sheet CO27-5).

### 2.3. Comparative Approach to Stwintron Verification

The coding sequences (ATG–stop) of the orthologue genes carrying [D1,2] stwintrons were mined in the *Hypoxylon* sp. CO27-5 genome as well as in other Hypoxylaceae genomes by TBLASTN screening of the NCBI’s Whole Genome Shotgun contigs (WGS) database [29] to track intron position conservation patterns as well as to appreciate the level of sequence conservation amongst position-conserved “orthologue” stwintrons found across the Hypoxylaceae. Twenty-two of the 23 full-length sister stwintrons defined in our previous work [25] occur only in *Hypoxylon* sp. CO27-5 and EC38, while two more sister stwintrons are unique to EC38 (i.e., these two are absent from CO27-5). The genome master accession numbers of the twenty fungi in the Hypoxylaceae family in which position-conserved [D1,2] stwintrons were encountered are listed and referenced in Table 1.

We manually predicted the intron–exon structure of each gene guided by comparative genomics of coding sequences, taking the intron phase into account. The sequence-derived information thus collected for each of the 117 *Hypoxylon* sp. CO27-5 [D1,2] stwintrons is shown in Supplementary Materials Table S1 (two Excel sheets). [D1,2] stwintrons found at positions that were not occupied in the *Hypoxylon* sp. CO27-5 orthologue genes (i.e., absent from CO27-5) were routinely collected from the other Hypoxylaceae genomes. The second sheet of Supplementary Materials Table S1, named “1413 [D1,2] stwintrons”, identifies the stwintrons encountered during the comparative analyses in the other genomes of the Hypoxylaceae family (19 taxa accessible at NCBI, excluding CO27-5 present on the first sheet), with their unique localisation given by the combination of the sequence contig in which the stwintron occurs and its exact coordinates (5′-GGY–AG-3′) therein.

Note that automated annotation software does not recognise [D1,2] stwintrons, complex intervening sequences that start with the noncanonical 5′-GGU (occasionally, 5′-GGC) at their 5′-splice site. We did not use the results of automated annotation (“Models” or “mRNA” in the nr/nt database), neither did we use deduced protein databases. After translation of our predicted mRNAs, we functionally annotated most of the genes carrying [D1,2] stwintrons with one or more protein domains categorised in the Protein Families (Pfam) database [36].

### 2.4. Other Informatics Methods Used

The sequence logo of the direct environment of the 5′- and 3′-stwintron/exon junctions in pre-mRNAs containing [D1,2] stwintrons was created by Skyline [37] using default settings. Sets of variables, collected in Supplementary Materials Table S1, for the 117 stwintrons identified in *Hypoxylon* sp. CO27-5, like (stw)intron lengths, stwintron phase, minimal free energy for the optimal predicted secondary structure and AU content were transferred to new spreadsheets for their individual analysis using the Excel tool box (Microsoft Office Professional Plus 2016; Microsoft Cooperation, Redmond, WA, USA), comparing the miscellaneous group of 81 “uniquely occurring” stwintrons (identified in the current work) with the group of the 38 sequence-similar sister stwintrons (cf., [25]). Multiple sequence Alignment with Fast Fourier Transform (MAFFT, version 7) [38] was used for the alignments of each single-stranded stwintron RNA sequence with its own reverse complement sequence, using E-INS-i iterative refinement. RNAfold [39,40] was used online (ViennaRNA web suite) with the default settings, except that isolated base pairs were not avoided, to predict the optimal secondary structure of single-stranded debranched stwintron RNAs, i.e., the structure with the lowest minimum free energy (ΔG). For each stwintron, the single optimal RNAfold structure was compared with the secondary structures proposed by another online predictor, Mfold (UNAfold web site) [41,42], which yielded multiple alternative structures with low predicted ΔGs. To improve the secondary structure prediction for the most sequence-similar (full-length) sister stwintrons, the sister stwintrons were first aligned by MAFFT, and then the alignment was trimmed manually by removing positions only occupied in one or two sister stwintrons. The optimal secondary structure of the 205 nt long “consensus” sister stwintron derived from the trimmed MAFFT alignment was predicted using RNAalifold (ViennaRNA web suite) [43].

### 2.5. Hypoxylon sp. CO27-5, Growth Medium and Nucleic Acid Isolation

A monoculture of *Hypoxylon* sp. CO27-5 (Ascomycota; Pezizomycotina; Sordariomycetes; Xylariales; Hypoxylaceae; *Hypoxylon*; unclassified *Hypoxylon*) was maintained on potato dextrose agar (PDA) (Neogen Culture Media, Lansing, MI, USA) [26]. For nucleic acid isolation, biomass was generated in submerged cultures in 100 mL potato dextrose broth (HiMedia Laboratories GmbH, Einhausen, Germany) in 500 mL Erlenmeyer flasks, in a rotary shaker (Infors HT Multitron, Infors AG, Lonay, Switzerland) at 200 rotations per min for 24 h at 25 °C. Cultures were inoculated with a dense suspension of mycelia, scraped from the surface of a one-week-old PDA seed plate (96 mm in diameter) in a sterile 1/10^4^ Tween-80 (VWR International LLC, Debrecen, Hungary) solution. Grown mycelia were harvested by filtration over Miracloth (Millipore, Merck KGaA, Darmstadt, Germany) and thoroughly rinsed with abundant sterile distilled water. Subsequently, the biomass was instantly deep frozen and ground to powder under liquid nitrogen using mortar and piston. Genomic DNA and total RNA were subsequently isolated using the Macherey–Nagel NucleoSpin Plant II and NucleoSpin RNA Plant kits, respectively (Macherey–Nagel GmbH & Co., KG, Düren, Germany).

### 2.6. Reverse Transcription Polymerase Chain Reaction (RT-PCR) and cDNA Sequencing to Validate Stwintrons

First strand cDNA was synthesised from total RNA template and Oligo(dT) as a primer using the RevertAid First Strand cDNA Synthesis Kit (Thermo Scientific, Thermo Fisher Scientific, Waltham, MA, USA). Using this single-stranded cDNA as the template, targeted PCR reactions were performed with gene-specific oligonucleotide primer pairs (Integrated DNA Technologies, Leuven, Belgium) (Supplementary Materials Table S2) and DreamTaq DNA Polymerase (Thermo Scientific, Thermo Fisher Scientific, Waltham, MA, USA) in a T100™ Thermal Cycler (Bio-Rad, Bio-Rad Hungary Ltd., Budapest, Hungary). Cycling conditions after initial denaturation at 95 °C for 2 min: 35 cycles of 95 °C for 30 s, 60 °C for 1 min and 72 °C for 0.5–1 min, followed by post-cyclic elongation at 72 °C for 5 min. Amplified DNAs were separated in native agarose gels (SeaKem LE Agarose; Lonza Group Ltd., Basel, Switzerland). All pairs of gene-specific PCR primers were verified with genomic DNA template for the amplification of the expected size, corresponding to the pre-mRNA sequence. To confirm the predicted stwintron splicing intermediate (splinter), primer pairs were designed that did not amplify DNA from fully spliced mRNA template. For [D] stwintrons, the gene-specific reverse primer hybridised entirely or partly (i.e., at its 3′ end) with a sequence within the external intron. When the target gene was expressed, this strategy almost always yielded two PCR fragments of which the smaller one corresponded to the stwintron splicing intermediate (splinter) and the larger one to the primary transcript (pre-mRNA). All experiments were conducted in duplicate, starting with the biomass from two independent liquid cultures (different inoculum). The cDNA of the expected size was gel-purified (NucleoSpin Gel & PCR Clean-up, Macherey- Nagel GmbH & Co., KG, Düren, Germany), then cloned in pGEM-T Easy (pGEM-T Easy Vector System I, Promega Corporation, Madison, WI, USA). Plasmid DNA was isolated using the NucleoSpin Plasmid EasyPure kit (Macherey–Nagel GmbH & Co., KG, Düren, Germany). Three independent clones were sequenced over both strands using universal primers hybridising to the vector (Eurofins Genomics, Ebersberg, Germany) or gene-specific oligonucleotide primers where appropriate. cDNA and splinter sequences from *Hypoxylon* sp. CO27-5 were deposited at GenBank under accession numbers: OL539745–OL539746, OL624519–OL624535, OL672706–OL672707, OM256448, OM541588–OM541592, OM719008–OM719015 and OM837808–OM837820.

## 3. Results and Discussion

### 3.1. The Hypoxylon sp. CO27-5 Genome Contains More Than 100 [D1,2] Stwintrons

In a previous communication, we identified a set of repetitive or multiple occurring (MO) intervening sequences, mostly [D1,2] stwintrons, in eight genomes of species of Hypoxylaceae and suggested how the most symmetrical canonical introns within this set in *Hypoxylon* sp. CO27-5 could be generated from an ancestor stwintron during microhomology-mediated end-joining (MMEJ) repair of a double-stranded DNA breaks [25]. These sequence-similar intervening sequences could be easily identified by BLASTN screening of the Whole Genome Shotgun contigs database (NCBI) using one of these intronic sequences of this group (HCOc017A) as the query sequence. During comparative studies, it became evident that genomes of the Hypoxylaceae species also included [D1,2] stwintrons seemingly unrelated to this set of 36 MO [D1,2] stwintrons, named sister stwintrons to reflect their sequence similarity and probable common ancestry. Two of the genes that contained a sequence-similar intervening sequence (i.e., HCOc091A and HCOc121A), incidentally, also carry a uniquely occurring (UO) [D1,2] stwintron that was not detected by the BLASTN screen (for cDNA sequences, see GenBank MW530491 and MW490721, respectively). Moreover, the gene encoding a DEXH-box DNA/RNA helicase in *Hypoxylon* sp. CO27-5, a fungal orthologue of the helicase domain of human polymerase theta (POLQ), also harbours a UO [D1,2] stwintron (for cDNA sequences, see GenBank MW530496).

To identify additional [D1,2] stwintrons in *Hypoxylon* sp. CO27-5, we analysed its genome sequences [26] with a sequence motif search algorithm that allowed for screening of the 580 contigs for putative stwintrons that are not sequence-similar to sister stwintrons (see Figure 2 and Section 2 (Materials and Methods) for more detail). The primary [D1,2] search model A identified 282 candidate sequences, which upon further analysis (see below) included 90 authentic [D1,2] stwintrons (>30% success rate), 59 (65%) of which were not identified by BLASTN screening with HCOc017A as the query sister stwintron. The 59 newly identified UO [D1,2]’s were numbered as the corresponding match numbers in the output of the [D1,2] search model A screening. Search model A picked up 21 of the 23 full-length sister stwintrons. HCOc224-179 could never be identified by a motif search approach, as it is split over two sequence contigs (see GenBank MW477887 for the whole sequence). The second sister stwintron missed by the primary [D1,2] search model A, HCOc021A, is a phase zero [D1,2] stwintron in a recently pseudogenised gene for a short-chain dehydrogenase/reductase that features a non-canonical 5′-donor at position 5 (5′-GUACC_5_UAUGU) for its internal intron in *Hypoxylon* sp. CO27-5 due to the presence of four extra nts near the 5′ end of the internal intron. These four nts are absent in strain EC38, where this 5′-donor is fully canonical (5′-GUAUGU), and the “orthologue” stwintron was detected by our primary [D1,2] search protocol when the EC38 genome was screened for [D1,2] stwintrons (n.b., the orthologous EC38 gene is also a pseudo gene).

In addition, three of the 13 sheared sister stwintrons (cf., [25]) were not recognised by the primary [D1,2] search model A screen. One of these is HCOc103A for which the internal intron distance between the donor–BP was 131 nt, much longer than the maximum setting in search model A (80 nt). In HCOc091A, the internal intron distance between the donor–BP was 82 nt, two nucleotides above the set limit. We adapted the search tool to screen for [D1,2] stwintrons in which the donor–BP distance in the internal intron would be between 81 and 110 nt (Figure 2, [D1,2] search model B) and found seven additional UO stwintrons, numbered no-315–321. Further increasing the donor–BP distance within the internal intron did not detect stwintrons other than HCOc103A.

By contrast, in HCOc263A, the distance between the donor-BP of the external intron was 126 nt, longer than the upper limit set in the primary [D1,2] search model A. The tool did identify the 5′ end of HCOc016B, but the canonical BP (motif 4) of the external intron of this stwintron associated with the functional 3′-acceptor was 8 nt above the set length range for the distance between the donor–BP; upon experimental verification (described below), the full-length stwintron was found to be 58 nt longer at 3′ than that proposed by the search tool using model A. These observations led us to change the distance range between the donor and the BP elements of the external intron from 25–110 to 111–200 nt in the [D1,2] stwintron search model (Figure 2, [D1,2] search model C). This resulted in the identification of another 13 UO [D1,2] stwintrons, numbered no-302–314. The largest stwintron found was 313 nt long (number no-313) while the shortest was 114 nt long (number no-061). Further increasing the distance range between the BP and its associated 3′-acceptor (for either U2 intron) from 4–20 to 21–40 nt did not lead to the identification of additional [D1,2] stwintrons.

Moreover, preliminary comparative analysis of two of the newly found [D1,2] stwintrons, numbers no-007 and no-061, showed that they are extant at the same position in two paralogue genes encoding short-chain dehydrogenases/reductases of 243 and 250 amino acids (AAs), respectively, ~54% identical at the AA level. Moreover, we found a third paralogue gene encoding a protein of 246 AAs, ~56% and ~59% identical to the two aforementioned paralogues, with a [D1,2] stwintron at the very same position. This latter stwintron (named number no-301) was not detected with the [D1,2] search model A, as the BP sequence of its external intron was the less conventional 5′-C_1_CTGAC, i.e., incompatible with the set motif for BP elements, 5′-D_1_YTRAY. We modified the stwintron search model in order to detect eventual additional [D1,2] stwintrons with a less canonical BP sequence (5′-C_1_CTRAY) (Figure 2, [D1,2] search model D) and found one additional [D1,2], number no-300, in the gene encoding an integral membrane protein of 318 AAs, with its external intron’s BP sequence 5′-C_1_CTAAC, canonical but for the nt at the first position.

### 3.2. Evidence for the Existence of 81 Predicted Uniquely Occurring [D1,2] Stwintrons

Next, we assessed all the sequence matches gathered with the subsequent [D1,2] stwintron model searches after subtracting the 31 matches corresponding to sister stwintrons, already experimentally validated in our previous paper [25]. We obtained evidence for the existence of an additional 81 UO [D1,2] stwintrons in *Hypoxylon* sp. CO27-5 using three approaches. The first approach involved a BLASTN search for short RNA sequence reads deposited in the Sequence Read Archives (SRA) database at NCBI (accessions: SRX875229–34; [26]), which confirmed the proposed exon fusion after the consecutive excision of the internal and external introns for each candidate [D1,2] as well as for reads that confirmed the existence of the typical stwintron splicing intermediate (abbreviated “splinter” below), the RNA species from which only the predicted internal intron was removed and which covered the temporary junction of the upstream exon and the now functional external intron before the excision of the latter and, ultimately, exon fusion. In Supplementary Materials Table S1 (sheet CO27-5), the SRA evidence for each of the 117 [D1,2] stwintrons identified in *Hypoxylon* sp. CO27-5 is represented by one of the covering SRAs. *Hypoxylon* sp. CO27-5 and EC38 are closely related [26]. When SRA reads were not found in CO27-5, we searched the accessible EC38 RNA SRA databases (SRX872662–67; [26]) for reads confirming complete removal of the “orthologue” [D1,2] stwintron as well as reads validating the predicted splinter.

Secondly, we assessed the relevant sequences of the fully spliced RNA species (mRNA) as well as the predicted splinter of selected [D1,2] stwintrons by targeted RT-PCR and cDNA sequencing, providing more sensitive means when the expression levels of the stwintron-containing gene are limiting. For the experimental details, see Section 2. The generated sequences were submitted to GenBank, and the accession numbers assigned are listed in Supplementary Materials Table S1 (sheet CO27-5).

Finally, we identified and collected the orthologues of the stwintron-containing CO27-5 gene in the genomes of 19 other species/strains of the Hypoxylaceae family accessible at NCBI (see Table 1) by targeted tblastn screening of the Whole Genome Shotgun (WGS) contig database (n.b., gDNA data). The comparative genomics approach enabled the confirmation of potential [D1,2] stwintrons, where expression data are insufficient or when gene expression cannot be detected by RT-PCR with pairs of gene-specific oligonucleotide primers functionally verified on genomic DNA as amplification templates. The intron–exon structures of these orthologue genes were manually predicted from the alignment of the coding sequences (exons) alternating with intronic sequences including the [D1,2] stwintron(s). Conservation of intron position(s) is considered a hallmark of orthologue genes (“intron positional conservation”; [44]) and, in some cases, of structurally related paralogue genes as well. By these means, we detected the occasional use of rare intronic splicing elements in [D1,2] stwintrons such as the occurrence of a C at the second position of 5′-donor elements (5′-GC_2_ instead of 5′-GU_2_) for either the internal or external intron, for instance, in the orthologue of stwintron number no-071 in *Entonaema liquescens* for the split external donor sequence (5′-G|C_2_AAGU).

In Supplementary Materials Table S1 (sheet CO27-5), we indicated the presence or absence of the “orthologue” intervening sequences for all 117 [D1,2] stwintrons now identified in *Hypoxylon* sp. CO27-5 or the absence of the orthologue gene in 16 columns near the right side of the Excel table. In the column at the right of Table S1, we indicated for most stwintron-containing genes whether there were structural paralogous genes of them in CO27-5; this was the case for more than half of the stwintron-harbouring genes (61) identified. In three cases—numbers no-007/no-061/no-301, numbers no-302/no-303 and sheared sister stwintrons HCOc020A/HCOc263A—it concerned paralogous genes with a position-conserved “paralogue” [D1,2] stwintron. In some other paralogous genes, [D1,2] stwintrons were identified in each, albeit not extant at the same intron position, e.g., for numbers no-158/no-189.

### 3.3. Occurrence of Occupied [D1,2] Stwintron Positions across Species of Hypoxylaceae

In our current work, we showed the existence of more than 1500 [D1,2] stwintrons in species of the Hypoxylaceae, a family of endophytic fungi in the Xylariales order of the Sordariomycetes class (Ascomycota phylum). The identification of [D1,2] stwintrons in *Hypoxylon* sp. CO27-5, experimentally or by comparative genomics, allowed, for the first time, for the statistical analysis of the sequences of more than one hundred different stwintrons of the same type present in one organism. Below, we compare a sizable group of sequence-related and terminally symmetrical stwintrons from *Hypoxylon* sp. CO27-5 and EC38, the 38 so-called sister stwintrons, with the 81 seemingly unrelated stwintrons identified with our [D1,2] stwintron search tool (see above).

**Figure 3:**
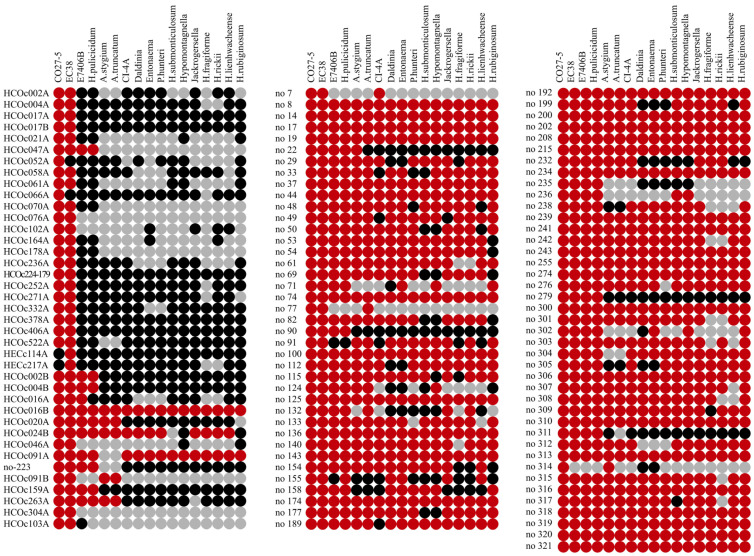
Occurrence of 117 orthologue [D1,2] stwintrons at the same gene position in 17 taxa of Hypoxylaceae. Four genomes in the *Daldinia* genus—*D. eschscholzii*, *Daldina* sp. EC12, *D. childiae* and *D. concentrica*—were considered as one taxon. The red circles indicate that the stwintron position confirmed in *Hypoxylon* sp. CO27-5 is also occupied by a [D1,2] stwintron in the intron–exon structure of the orthologue gene in another species or strains of Hypoxylaceae. The black circles indicate that the CO27-5 stwintron position was not occupied in the orthologue gene in another species or strains. The grey circles indicate the absence of the orthologue gene in other species or strains. Twenty-four of the 25 full sister stwintrons (named as in [25]) exclusively occurred in the strongly related strains CO27-5 and/or EC38 with four sister stwintrons unique to either CO27-5 or EC38. For almost all of the 81 newly reported, uniquely occurring (UO) stwintrons, occupation of the stwintron position was not restricted to *Hypoxylon* sp. CO27-5 or EC38. Note that for stwintron number no-274, the [D1,2] stwintron changed to the [D5,6] configuration at the very same position in *H. rickii*, *H. fragiforme* and *H. lienhwacheense* (see Section 3). The genome resources used in this work are identified by their unique master genome accession number in Table 1 along with the original literature reporting on these resources. All genomes are freely accessible at NCBI.

The comparative approach immediately showed a substantial distinction between these two groups (Figure 3). While 24 of the 25 sister stwintrons only occurred in the close relatives of *Hypoxylon* sp. CO27-5 and/or EC38 (n.b., four of them are unique to either CO27-5 or EC38), the large majority of the UO [D1,2] stwintrons (68 out of 81: ~84%) were present in orthologue genes in all or most of the 17 assessed species/taxa of the Hypoxylaceae family. Amongst the 13 “atypical” UO stwintrons, five were located in genes that did not have orthologues in most of the other assessed species/taxa. These large differences in the occurrence of orthologue stwintrons (i.e., at conserved gene positions) strongly suggest that the large majority of the group of 81 UO stwintrons are much older than the highly symmetrical sister stwintrons unique to the CO27-5/EC38 taxon, as the former were likely generated in a common ancestor before divergence of all 20 taxa. The group of the 13 sheared sister stwintrons appears to be composed of mixed constituents: six of them were restricted to a narrow taxonomical clade that consists of or includes both *Hypoxylon* sp. CO27-5/EC38 and *H. pulicicidum*/*Hypoxylon* sp. E7406B.

Three other sheared sister stwintrons occurred only in CO27-5/EC38, because there are no orthologue genes in the other taxa (not including *H. pulicicidum*/E7406B). On the other hand, three sheared sister stwintrons identified by BLASTN screening with HCOc017A—HCOc016B, HCOc024B and HCOc091A (cf., [25])—were present at a conserved position across the whole family and would appear to be misclassified as sheared sister stwintrons. In our previous work, we noted that there are also ten genuine sister stwintrons of the same pedigree/origin in the *H. pulicicidum*/E7406B taxon that are absent from the CO27-5/EC38 taxon [25], which supports the idea that the MO and terminal symmetrical sister stwintrons were recently generated.

A peculiar situation was observed for the phase-two stwintron number no-274 in a gene encoding a well-conserved plasma membrane protein of the monovalent cation:proton antiporter-1 family, suggesting that extant [D] stwintrons may change stwintron type by local mutation. In three species—*H. rickii*, *H. fragiforme* and *H. lienhwacheense*—the original [D1,2] stwintron found in the other Hypoxylaceae species and also present in other Xylariales species presumably morphed into a [D5,6] stwintron. Insertion of four nt 5′-GTAA or 5′-GTGA directly upstream of the G_1_ of the external intron of the original [D1,2] stwintron created (split) external intron donors 5′-GTAAG_5_|T_6_ or 5′-GTGAG_5_|T_6_ (Supplementary Materials Figure S1). The internal intron and the bordering exons were unaffected, as the stwintron phase did not change. In *H. fragiforme*, the loss of the [D1,2] splice option was the result of a 20 nt deletion in the 5′-sequence of the external intron directly downstream of the junction with the internal intron (5′-TAG|T_2_).

### 3.4. No Insertion Site Bias Observed for Exonic Sequences Directly Neighbouring [D1,2] Stwintrons

**Figure 4:**
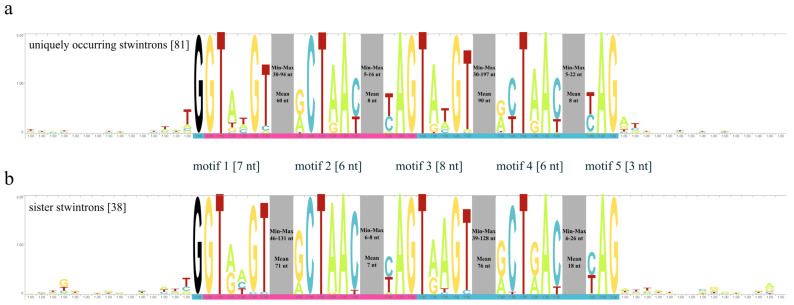
Sequence Logo of the stwintron–exon junctions of the 81 uniquely occurring [D1,2] stwintrons in *Hypoxylon* sp. CO27-5 compared with that of the group of 38 full-length sister and sheared sister stwintrons described in [25]. (**a**) The Logo for the group of newly described, uniquely occurring (UO) stwintrons (81 sequences) without sequence similarity of note, while (**b**) shows the Logo for the sequence-related sister stwintrons (38 sequences, including two EC38-specific sister stwintrons). Logos visualise the extent of nt conservation in multiple sequence alignments and highlight patterns of conservation. The core of the multiple sequence alignments consists of the concatenated sequences of the five consensus sequence motifs at the splice sites and the canonical BP sequences of the internal and external introns within the [D1,2] stwintron (together 30 nt: see also Figure 2). At each end of the five concatenated stwintron sequence motifs, the 15 nt of the exonic sequences directly bordering the stwintron were added to the input DNA. The sequences of the internal intron are indicated by the magenta bar underneath the Logo, and the sequences of the external intron are indicated by the turquoise bar. The ubiquitous black G (position 16) is the G_1_ of the split donor of the external intron of the stwintrons in [D1,2] sequence motif 1. The four spacer sequences between the five conserved [D1,2] sequence motifs are not drawn to scale; these sequences lack similarity amongst the 81 UO stwintrons. The spaces (grey boxes) were used to summarise details about the length of the spacers between the donor and canonical BP sequence elements, and the length of the spacers between canonical BP sequence elements and the acceptors (i.e., the minimum, maximum and mean length) of these four intronic spacer sequences are given.

We analysed the insertion sites of the 81 MO [D1,2] stwintrons after stacking the five concatenated conserved sequence motives at the 5′- and 3′-splice sites (donor; BP; acceptor; including the two hybrid motifs typical for the [D1,2] type) terminally extended with the first 15 nt of their neighbouring exons and compared with the local exon environments with the same Logo analysis for the “control” group of 38 sister stwintrons (Figure 4).

The insertion sites of stwintrons of both groups are seemingly not sequence biased; there were no conserved sequence patterns in the exons directly next to the stwintron–exon junctions. In line with the above, there was no prevalence for alternatively spliced [D1,2]/[A2,3] stwintrons (i.e., a G directly downstream of the [D1,2] stwintron) (Supplementary Materials Figure S2). The combination of [AG] directly upstream of the stwintron and [GT] directly downstream of it—comparable with double AG|GT fusion sites for certain canonical introns (e.g., [45])—was not found in our set of 117 stwintrons; on probability grounds, one would expect this to occur once in every 256 cases. Furthermore, there was no evidence for tandem site duplication involving one of the splice sites (cf., [46]) in the *Hypoxylon* sp. CO27-5 stwintrons. There were two instances of [TAG] directly upstream of a stwintron terminating with the 3′-acceptor TAG, but [CAG] did not occur directly upstream of any of the *Hypoxylon* sp. CO27-5 stwintrons.

Meanwhile, [GT] directly downstream of most of the 3′-acceptors of the stwintron occurred in eight of the 117 stwintrons, while [GTA] was present in two of them (i.e., numbers no-279 and no-308).

All 81 UO stwintrons were seamlessly integrated in continuous exonic sequences, without loss or gain of exonic sequences, very much as it was observed for all but one of the sister stwintrons (exception described in [25]). Their exact position and phase were conserved amongst orthologous stwintrons in other Hypoxylaceae species. There were considerably more phase one stwintrons (~45%) than there were phase two or phase zero stwintrons, but also in this respect, the profile was very similar for both groups of [D1,2] stwintrons (Supplementary Materials Figure S2).

### 3.5. The Distance between the Lariat Branch Point Sequence and the Acceptor of the External Intron Was Twice as Long in the Sister Stwintrons as in the Uniquely Occurring, Evolutionary Older Stwintrons

Collemare and co-workers [47] formulated a hypothesis by which most conventional U2 introns (“regular spliceosomal introns” or RSIs) currently present in filamentous fungal genomes derive from ancestor introner-like elements (i.e., highly sequence-similar introns with stable secondary structures) by propagation events that have taken place so long ago that the duplicated intronic sequences have diverged beyond recognition. Degeneration of introner-like elements would not only lead to rapid sequence divergence but also to a consistent shortening of U2 introns towards the mean length of regular spliceosomal introns (cf., [47]).

When the length distribution of the CO27-5 stwintrons was assessed, together with the lengths of the constituent canonical introns (Supplementary Materials Figure S3), we found that the range of intron sizes for the group of the older UO stwintrons was much broader than those for the sister stwintrons. Such a result was not unexpected, as sister stwintrons share considerable sequence similarity and appear to have been generated recently after the divergence of *Hypoxylon* sp. CO27-5/EC38 and *Hypoxylon* sp. E7406B/*H. pulicicidum* (cf., [25]). Their relatedness is also reflected by the peaks in lengths for the sister stwintrons and their respective internal and external introns in the histograms’ moving averages (red line in Supplementary Materials Figure S3a). Amongst the miscellaneous group of 81 UO stwintrons, there are 13 stwintrons that were, indeed, shorter than any sister stwintron, but ten others were longer than 37 of the 38 sister stwintrons. The shorter UO stwintrons seemed to coincide with “shrunken” internal introns, while in the longer UO stwintrons, it was the external intron that increased in size in most cases.

In general, the differences in stwintron size were confined to the core of the constituent intron between the 5′-donor and the lariat branch point sequence (BP). By contrast, we observed a proclivity to conserve a relatively short distance between the canonical branch point sequence (BP) and (associated) 3′-acceptor for both the internal and the external intron in the older UO stwintrons. The distance between the canonical lariat branch point sequence (BP) and 3′-acceptor of the internal intron was seven nucleotides in 34 of the 38 sister stwintrons (89%) as well as in 49 of the 81 UO stwintrons (60%) along with 11 other stwintrons with this distance being six nucleotides and five other stwintrons with this distance being eight nucleotides (Figure 4). For the external intron, the distance between the canonical BP element and the 3′- acceptor was six nucleotides in 50 of the 81 UO stwintrons (~62%), while in a further six of these, the distance was seven nucleotides, and in three others, the distance was five nucleotides. However, in the sister stwintrons, the distance between the canonical BP sequence and the 3′-acceptor of the external intron is typically 19 nt, found in 23 of the 25 full sister stwintrons (92%) and 5 of the 13 sheared sister stwintrons, while 3 other sheared sister stwintrons had that distance at 18 nt. On the other hand, there were two sheared sister stwintrons—HCOc016B and HCOc024B—in which the distance between the canonical BP element and acceptor was six nucleotides, perhaps, not coincidently, by far the most frequently occurring distance in the miscellaneous group of UO stwintrons.

In animals, the U2 snRNP auxiliary factor (U2AF protein) recognises the pyrimidine (CU) tract and the downstream 3′-splice site, and then interacts with the U2 snRNP upon which the latter is recruited to face the functional lariat branch point sequence in the assembling U2 spliceosome (reviews: [8,9]). Genes for both subunits U2AF65 [contig MDCL01000013, coordinates c90609–88714] and U2AF35 [contig MDCL01000186, coordinates 104888–105517] are present in *Hypoxylon* sp. CO27-5 (not shown). In line with what has been observed in model ascomycete fungi [27], where pyrimidine tracts occur, these are usually situated 5′ to the canonical BP sequence, often closer to the 5′-donor than to the BP element. Such location is logical because the distances between the canonical BP elements and the functional 3′-acceptors are shorter than 8 nt in the large majority of the UO (older) stwintrons. Remarkably, we observe that in the external intron of the 25 full-length sister stwintrons in our set, uninterrupted pyrimidine (CT/CU) tracts longer than 6 nt occur sporadically (only in HCOc021A & HCOc047A). By contrast, half of the sheared sister stwintrons do have sizable pyrimidine tracts between the 5′-donor and the canonical BP element of the external intron, while two of them—HCOc046A and HCOc263A—have a pyrimidine tract (>7 nt) downstream of the canonical BP element of the external intron. In the set of miscellaneous UO stwintrons, most external introns feature clear pyrimidine tracts between donor and canonical BP sequence. In stwintrons numbers no-071 and no-274, a pyrimidine tract (>7 nt) is identifiable between the canonical BP sequence and the downstrean acceptor of the external intron; It concerns two of the older UO stwintrons where the distance between these consensus intron sequence elements is considerably longer (17 nt and 15 nt, respectively) than the mean size (six nt, see above).

### 3.6. The AU Content in the Propagating Sister Stwintrons Is Higher Than in the Uniquely Occurring Stwintrons

**Figure 5:**
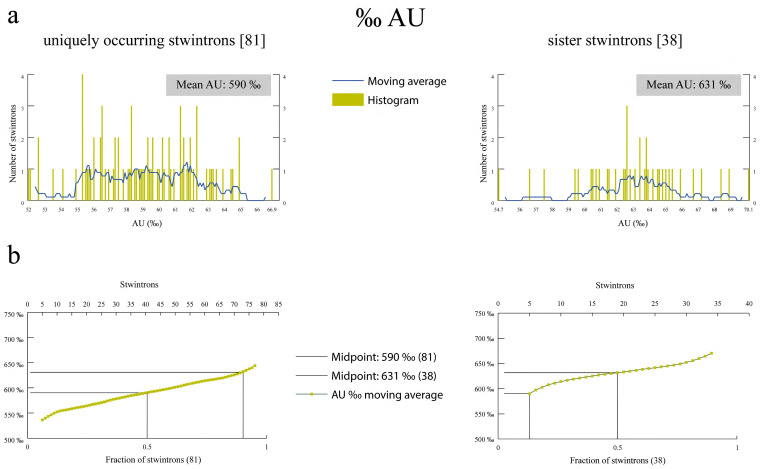
The AU content of sister stwintrons is consistently higher than that in the miscellaneous group of evolutionary older, uniquely occurring stwintrons. (**a**) shows the crude data in histogram form specifying the number of stwintrons with one particular AU content rounded to units permille (‰). A moving average of the AU content over a window of nine consecutive permillages (number of AU/number of nt for each stwintron × 1000) was calculated for single step increments in AU content (blue line). In (**b**), incrementing moving averages of AU content (green data points) were plotted against the fraction of stwintrons for each of the two groups. The mean AU content in each group of stwintrons (permillage AU at stwintron fraction 0.5) was indicated with horizontal and vertical lines in both panels.

Without exception, the 117 *Hypoxylon* sp. CO27-5 [D1,2] stwintrons contain more AU than GC (Figure 5); AU content varies between 520‰ and 701‰ AU (Supplementary Materials Table S1). When the average value over nine consecutive AU‰ is plotted against the stwintrons ordered to increasing AU‰ (Figure 5), we observe that in the group of the sequence-similar sister stwintrons the ‰ AU is considerably higher, mean value 631‰, than in the miscellaneous group of 81 UO stwintrons, where the mean value is 590‰. In fact, all 25 full-length sister stwintrons in our set have over 600‰ AU content. At variance with this, nine of the 81 UO stwintrons have an AU‰ higher than the mean value in the group of 38 sister stwintrons.

### 3.7. All 117 [D1,2] Stwintrons Show Underlying Symmetry

**Figure 6:**
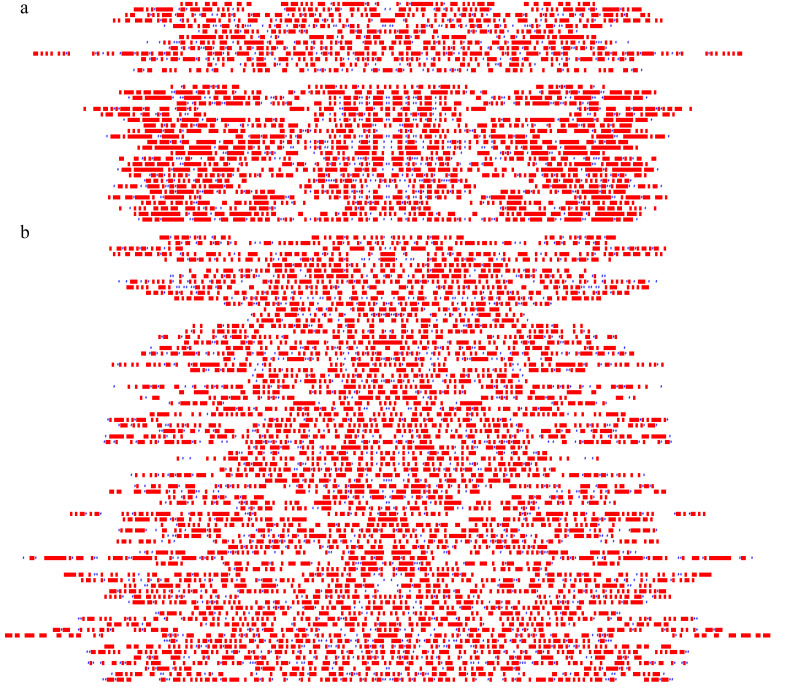
All *Hypoxylon* sp. CO27-5 stwintrons exhibited an underlying internal symmetry. (**a**) A two-dimensionally horizontal schematic representation of internal symmetry is shown for each of the 38 sequence-similar sister stwintrons, the 13 sheared sister stwintron at the top and the 25 full-length sister stwintrons below with the two unique EC38 sister stwintrons at the bottom. The stwintrons are vertically ordered as the order in which they appear in Supplementary Materials Table S1. The midpoints of the symmetry, as indicated by the MAFFT alignment of each stwintron sequence with its own reverse complement sequence, are aligned at the centre of the figure, resulting in a visual effect where the left and right half of the panel are mirrored. (**b**) The same representation of the internal symmetry in each of the uniquely occurring (evolutionary older) [D1,2] stwintrons. The 81 stwintrons are vertically ordered by increasing the match number of the stwintron amongst the hits scored in the primary search model for [D1,2] stwintrons (i.e., the order in which they appear in Supplementary Materials Table S1). The schematic representation of internal symmetry was derived from the one-on-one alignment of a stwintron sequence with its own reverse complement sequence by MAFFT using the E-INS-I module and employing PAM matrices to reduce the number of gaps introduced and limiting the extent of these gaps. A red square represents positions in the alignment where the nt (irrespective of its identity) in the stwintron sequence was the same as in its reverse complement sequence. The blue # (number) sign represents positions in the alignment where noncanonical GU or UG base pairing can occur in double-stranded sections. In all other situations, the position was left white in the graphic two-dimensional scheme of the alignment. Different alignment methods sometimes yield symmetries with a different midpoint for one and the same stwintron. This figure shows only one scheme per stwintron, the scheme representing the most parsimonious symmetry, i.e., with the least gaps introduced in the MAFFT alignment and the smallest possible gap length/extent. The MAFFT alignment was carried out so that, in most cases, it resulted in imperfect symmetries over the whole width of the stwintron or with a 5′ or 3′ overhang. This suggests that the stwintron can fold into one imperfect hairpin structure (see, e.g., Supplementary Materials Figure S4). Nevertheless, such hairpins may not be the optimal secondary structure corresponding to the lowest minimum free energy of the stwintron RNA.

In our previous communication, we noticed the extraordinary symmetry of some canonical U2 introns in *Hypoxylon* sp. CO27-5 with high sequence similarity to sister stwintrons, hinging in the centre of a 10-nt palindrome 5′-TTTCTAGAAA ([25]: cf., Figure 6a therein) and predicted to fold into one hairpin secondary structure leaving the unpaired 5′-G_1_ of the donor and the 3′-G_3_ of the acceptor in very close proximity. These so-called type-2 cropped sister introns most likely evolved recently from an ancestor sister stwintron with two internal palindromic sequences 5′-WTTCTAGAAA separated by approximately 100 nt, when a double-stranded DNA break between the two palindromes would have been repaired by microhomology-mediated end-joining. Some 40% of the 23 sister stwintrons of high sequence similarity indeed exhibited terminal inverted repeats 45–55 nt in length with 65–78% identity among them, when the stwintron RNA sequence was aligned with its own reverse complement sequence under stringent conditions considering the introduction of gaps as means to increase the identity score.

We studied internal symmetry with the same simple means (see above) in the 81 UO stwintrons newly identified and found that without exception, some level of sequence symmetry could be detected (Figure 6: panel a, the “control” group of 38 sister stwintrons; panel b, the 81 UO stwintrons). Using different alignment programs, often two different centres of symmetry were apparent in the same stwintron. This could be a reflection of the dynamics of RNA secondary structure, locating different or alternative internal stem-loop structures, either mutually exclusive or concurrent, in the core of the intervening sequence.

Almost all of the symmetries shown in Figure 6 result from MAFFT alignments employing the E-INS-i module while using either the 200 PAM, 20 PAM or 1 PAM scoring matrix to minimise the number of gaps introduced and to reduce the extent of those introduced gaps in the alignment of the stwintron RNA with its own reverse complement sequence. For most of the full-length sister stwintrons (19 from 25), internal symmetry is particularly dense near the termini of the stwintron while this is also the case in two sheared sister stwintrons, HCOc004B and HCOc046A. In the miscellaneous group of older UO stwintrons, MAFFT often produced alignments with a long overhang at either the 5′-terminus or the 3′-terminus, and also alignments with huge gaps, suggestive of big deletion or insertion events at the DNA level, unmatched at the opposite half of the stwintron. Interestingly, in two stwintrons—numbers no-215 and no-319—the terminal overhang is so long that the MAFFT alignment in effect suggests the existence of a symmetrical element that essentially corresponds exclusively to the external intron of the stwintron. Nevertheless, there are at least 30 UO stwintrons that produce continuous or near-continuous sequence alignments of the stwintron RNA with its own reverse complement sequence, with no or few small gaps near the termini and modest terminal overhangs (ten examples in Supplementary Materials Figure S4). This underlying symmetry may no longer be involved in RNA structure dynamics but could be a vestige of more extended or stonger ancestral terminal inverted repeats. Our observations of symmetry in all 117 *Hypoxylon* sp. CO27-5 [D1,2] stwintrons may be taken as an indication in support of the thesis of Collemare and co-workers [47] that many “regular spliceosomal introns” (RSI) present today in filamentous fungal genomes actually descend from ancient introner-like elements, repetitive intronic sequences that were predicted to fold in a characteristic hairpin secondary structure, and capable of intron proliferation. But the question remains to what extent the underlying internal symmetry of the complex intervening sequences in *Hypoxylon* sp. CO27-5 is inconsequential—all single-stranded RNA molecules showed secondary structure dynamics—and at what level those alternating secondary structures could influence the splicing process, to shift between the alternative splicing options and therewith participate in (stw)intron propagation.

### 3.8. Secondary Structure Predictions of Hypoxylon sp. CO27-5 [D1,2] Stwintrons

A new class of spliceosomal introns was reported in zebrafish (*Danio rerio*) several years ago, where splicing depends on the intron’s RNA secondary structure [48,49,50]. Splice-site pairing concurs with the presence of repeats of complementary dimers AC near the 5′-donor and matching GU couples near the 3′-acceptor, allowing the single-stranded intron RNA to fold across the intron bringing its splice sites in close proximity. Using RNAfold software [39,40], the optimal predicted secondary structures of the single-stranded RNA of these so-called (AC)m-(GU)n introns reportedly have minimum free energies (ΔG) up to two-fold lower than those calculated for conventional U2 introns of the same length in zebrafish, over a broad range of intron lengths. Remarkably, (AC)m-(GU)n introns are spliced out without the involvement of the U2 snRNP auxiliary factor (U2AF protein: 2 subunits) necessary to recruit the U2 snRNP to the functional branch point sequence of conventional U2 introns. The RNAfold-predicted secondary structure of an exemplary (AC)m-(GU)n intron (i.e., intron 5 of the *D. rerio* cep97 gene for a centrosomal protein—accession number: NC_007112, coordinates 13333–13503) (cf., [48]) can be described as one extended albeit imperfect hairpin leaving the terminal G’s unpaired but in close proximity to each other. Conserved features, such as the branch point element, are masked in double-stranded sections in the imperfect hairpin structure.

This zebrafish’s optimal RNA structure bears a cunning resemblance to the RNAfold-predicted structures of half of our type-2 cropped sister introns in *Hypoxylon* sp. CO27-5 as well as with those of some of the 25 full-length sister stwintrons (Supplementary Materials Figure S5, bottom row), in particular with respect to what we previously defined as terminal inverted repeats in the sister stwintrons and the type-2 cropped sister introns (cf., [25]). The canonical BP elements of the external introns of many sister stwintrons were partially or completely masked from the spliceosome in one of the double-stranded sections of the terminal stem structure However, stretches of complementary dinucleotide repeats (AC–GU) near the opposite termini did not appear in any of the 38 *Hypoxylon* sp. CO27-5 sister stwintrons or in any of the ten type-2 cropped sister introns (and neither in the group of the 81 UO stwintrons).

We assessed the minimum free energies (ΔG) for the optimal secondary structures in our set of 117 stwintrons according to the RNAfold predictor to estimate whether the ΔG provides another distinction between the sister stwintrons (the “younger” stwintrons) and the miscellaneous group of 81 UO stwintrons (the “older” stwintrons) (Figure S6).When the predicted ΔG values (listed in Supplementary Materials Table S1) were plotted against the fraction of stwintrons ordered to increasing size, the data points were scattered around the plot. We resorted to linear regression of each of the two data sets in the size range defined by 37 sister stwintrons, from 160 to 226 nt in length, to identify trends for each group of stwintrons. The two fitted lines suggested that the minimum free energies were on average, some 18–20% lower for sister stwintrons than for the UO stwintrons in the same size range, with many individual exceptions in both sets of stwintrons. These differences in ΔG are thus less pronounced for (AC)m-(GU)n introns versus conventional U2 introns in zebrafish (50–100% lower) [48].

**Figure 7:**
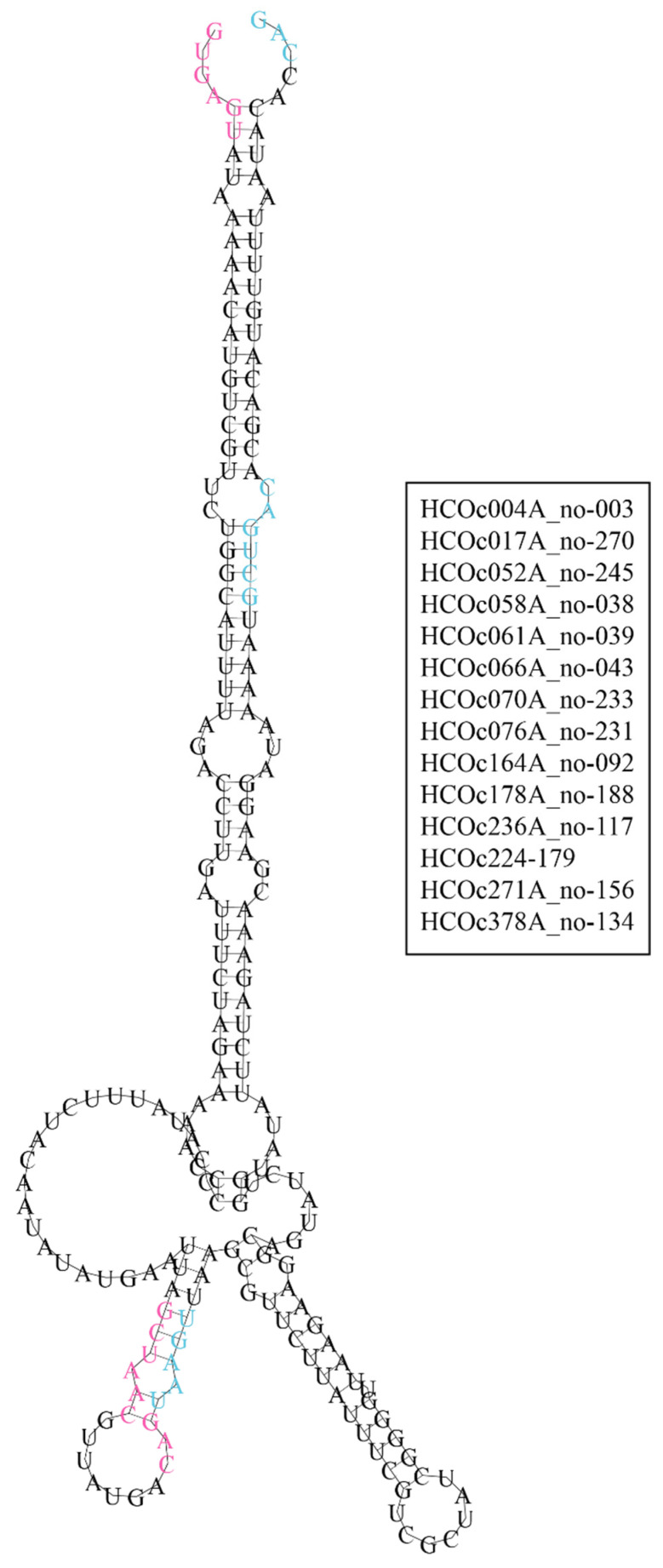
Optimal secondary structure prediction of a consensus sister stwintron deduced from a multiple sequence alignment. The RNAalifold program predicts the folding of a consensus RNA sequence derived from a Multiple Sequence Alignment of a set of sequence-similar RNAs. The basis of the Figure is the MAFFT alignment of the 23 *Hypoxylon* sp. CO27-5 sister stwintrons shown in our previous publication (cf., [25], Figure 2 therein). Two of the most sequence divergent stwintrons (i.e., HCOc002A and HCOc552A) were omitted. Positions where nts were present in only one or two of the sequences were eliminated from the alignment. Base deletions specific to some of the stwintrons were addressed: seven additional sister stwintrons were excluded from the alignment as they present single gaps bigger than 3 nt. The resulting “consensus” sister stwintron was 205 nt in length and based on the 14 most similar sister stwintrons (listed in the inlet at the right). RNAalifold (default settings, except that isolated base pairs were not avoided) predicted the secondary structure of this consensus [D1,2] sister stwintron. The canonical sequences of the conserved 5’-donor, BP sequence element, and 3’-acceptor of its internal intron are printed in magenta letters. The canonical sequences of the conserved 5’-donor—split by the internal intron between G_1_ and U_2_—BP sequence element, and 3’-acceptor of its external intron are printed in turquoise letters.The minimum free energy ΔG for this folding was –68.71 kcal/mol (–27.16 kcal/mol from covariance contributions).

To improve the reliability of the secondary structure prediction, the 14 most similar sister stwintrons were aligned before folding was predicted for a “consensus” sister stwintron deduced from the alignment. Figure 7 shows the secondary structure prediction of a parsimonious consensus sister stwintron (length 205 nt) using the RNAalifold program from the online ViennaRNA suite [43]. A stem-loop structure was predicted, where the stem is formed by the terminal inverted repeat (cf., [25]); this “consensus stem” is 47 nt long from which 8 nt are not base paired, without bulges but including two noncanonical GU base pairs. The canonical BP sequence of the external intron was masked by a double-stranded section of the consensus stem. In the centre of the consensus structure two small hairpins were predicted, neighbouring each other in this most parsimonious structure. Interestingly, in the most 5′ of these predicted small hairpins, the nine-nucleotide-long stem comprised the six-nucleotide canonical BP element of the internal intron (5′-GCUAAC) base paired with the six-nucleotide donor element of the external intron (5′-GUAAGU) (one mismatch), with the branch point A_5_ and the donor G_1_ (functionally for normal stwintron excision, the 3′-G_3_ of the internal acceptor) locked in neighbouring base pairs. It would thus appear that in the predicted consensus sister stwintron structure (RNAalifold), the internal splice elements mask each other from the spliceosome RNP. The consensus sister stwintron did not contain sequences of more than five consecutive pyrimidines, but an interrupted tract (5′-UUCUUAUUUC) was (exactly) locked in the stem of the 3′ of the small hairpins near the centre of the consensus structure (Figure 7).

The zebrafish (AC)m-(GT)n introns are spliceosomal introns with secondary structures that appear to be under positive selective pressure to guarantee their proper excision [48]. However, the existence of strong hairpin-like or stem-loop structures in *Hypoxylon* [D1,2] sister stwintrons potentially driving the pairing of distal splice sites and subsequent excision of (almost) the complex intervening sequence (minus G_1_) in one reaction presents a contradiction with the typical two-step mode by which stwintrons are excised and which we showed to overwhelmingly occur in the set of 117 [D1,2] stwintrons (Supplementary Materials Table S1). By definition for any stwintron, exact removal of the whole complex intervening sequence is only possible with two consecutive standard U2 splicing reactions, where the excision of the internal canonical U2 intron necessarily precedes the excision of the external canonical U2 intron (cf., [18]). Immediate utilisation of the most distal splice sites of a [D1,2] stwintron by the spliceosome, in effect an alternative excision by one standard splicing reaction, leads to a +1 frameshift at the exon-exon junction and in most cases, in C-terminally truncated protein products. The RNA species that forms after such a mis-splicing event is usually turned over rapidly by means of nonsense-mediated mRNA decay (for reviews on nonsense-mediated mRNA decay, see, for example [51,52,53,54]). Nevertheless, we were able to find SRA reads that proved that this alternative splicing of [D1,2] stwintrons in one standard splicing reaction does occasionally occur in *Hypoxylon* sp. CO27-5, notably for both sister stwintrons and UO stwintrons (Supplementary Materials Table S3).

It would therefore appear that splice-site pairing of the internal intron of the *Hypoxylon* sister stwintrons by intron definition is by far prevalent over splice-site pairing mediated by the secondary structure of the complete intervening sequence. The exact removal of the stwintron sequence by two consecutive conventional splicing reactions would occur despite the remarkable observation that the internal splice sites of sister stwintrons could mask each other from recognition by the assembling spliceosome. This prevalence would imply that in evolutionary older UO stwintrons that occur in most sequenced species of the Hypoxylaceae family (i.e., the group of 81), the secondary structure of the whole intervening sequence is not conserved between species, because the selective pressure bears solely on the correct excision by two consecutive standard U2 splicing reactions. We tested this in nine [D1,2] stwintrons that were position-conserved in most species of the family (HCOc024B and numbers no-053, no-054, no-082, no-143, no-208, no-223, no-239 and no-301; their predicted optimal secondary structures in *Hypoxylon* sp. CO27-5 are shown in Supplementary Materials Figure S5) and found no evidence for the conservation of the optimal secondary structure as predicted by RNAfold, not even in *Hypoxylon* sp. CO27-5 and the closely related species *Hypoxylon pulicicidum*.

## 4. Conclusions

For the first time, the existence of stwintrons of nested canonical U2 introns could be studied, analysing more than one hundred [D1,2] stwintrons present in one fungus, *Hypoxylon* sp. CO27-5. Most members of the group of the 81 uniquely occurring stwintrons were conceivably older than the sequence-related sister stwintrons exclusive to *Hypoxylon* sp. CO27-5 and EC38, as the former occur at conserved gene positions also occupied by [D1,2] stwintrons in other species of the Hypoxylaceae family. The two groups of stwintrons have some common characteristics, notably the common absence of exonic sequence bias at the exon–stwintron junctions, the seamless insertion in previously continuous exons and a very similar phase distribution with a clear predilection for phase one. The AU content and the presence of intronic pyrimidine tracts were clearly different when comparing the two groups of [D1,2] stwintrons. Remarkably, the distance between the canonical BP element and its associated 3′-acceptor was three times longer in the external intron of the 23 full-length sister stwintrons (19 nt) than in the external intron of most of the 81 older stwintrons (5–7 nt in 75% of the latter), albeit essentially similarly short as the latter for the internal introns of both groups of stwintrons. The presence of an underlying symmetry in all 117 stwintrons is surprising, although the symmetry is more pronounced near the extremities of most of the 23 full-length sister stwintrons. This terminal inverted repeat has the potential to form an extensive secondary structure that brings in close proximity the stwintron’s most distal 5′- and 3′-splice sites. This raises the prospect of secondary structure-mediated excision of almost the whole stwintron sequence in one reaction. However, the *Hypoxylon* stwintrons are overwhelmingly excised by consecutive splicing reactions that precisely remove the intervening sequence, rather than being engaged in one splicing event that creates a frameshift (+1) in the joined exons. Indeed, SRA evidence (cf., Supplementary Materials Table S3) suggest that the latter alternative (mis)splicing takes place for both the recent sister stwintrons and the evolutionary older uniquely occurring stwintrons. The internal symmetry of sister stwintrons thus seems dispensable for the rare excision between the distal splice sites and its involvement in sister stwintron propagation more likely concerns the stability of the folded excised stwintron RNA.

## Figures and Tables

**Table 1:** Genome and SRA resources employed in this work.

Organism	WGS Master Accession	SRA Accessions (RNA)
*Hypoxylon* sp. CO27-5	MDCL00000000 [26]	SRX875229–SRX875234 [26]
*Hypoxylon* sp. EC38	MDCK00000000 [26]	SRX872662–SRX872667 [26]
*Hypoxylon* sp. E7406B	JYCQ00000000 [30]	
*Hypoxylon pulicicidum* ATCC 74245	PDUJ00000000 [31] CADCWX000000000 [32]	
*Hypoxylon* sp. CI-4A	MDGY00000000 [26]	
*Annulohypoxylon stygium* MG137	PYLT00000000 QLPL00000000 [33]	
*Hypoxylon rubiginosum* MUCL 52887	CADCXA000000000 [32]	
*Daldinia* sp. EC12	MDGZ00000000 [26]	
*Daldinia eschscholzii* IFB-TL01	AKGB00000000 [34]	
*Daldinia childiae* JS-1345	VYXO00000000 [35]	
*Daldinia concentrica* CBS 113277	CADCSW000000000 [32]	
*Entonaema liquescens* ATCC 46302	CADCSX000000000 [32]	
*Hypomontagnella monticulosa* MUCL 54604	CADCWR000000000 [32]	
*Hypoxylon fragiforme* MUCL 51264	CADCWU000000000 [32]	
*Hypomontagnella submonticulosa* (*Hypoxylon submonticulosum*)	CADCWV000000000 [32]	
*Hypoxylon lienhwacheense* MFLUCC 14-1231	CADCWW000000000 [32]	
*Pyrenopolyporus hunteri* MUCL 49339	CADCXC000000000 [32]	
*Annulohypoxylon truncatum* CBS 140778	CADCSV000000000 [32]	
*Hypoxylon rickii* MUCL 53309	CADCWY000000000 [32]	
*Jackrogersella multiformis* CBS 119016	CADCXD000000000 [32]	

## Data Availability

Data are contained within the article and the associated Supplementary Materials. Accession numbers for sequences determined during this study: GenBank OL539745–OL539746, OL624519–OL624535, OL672706–OL672707, OM256448, OM541588–OM541592, OM719008–OM719015 and OM837808–OM837820.

## Supplementary Materials

The following supporting information can be downloaded at: https://www.mdpi.com/article/10.3390/jof8040397/s1. Figure S1: Sequence alignment of stwintron number no-274 in orthologue genes for a monovalent cation:proton antiporter in species of Xylariales (Hypoxylaceae, Xylariaceae and other families); Figure S2: percentages of overlapping [D1,2] and [A2,3] stwintrons, and of stwintron phases; Figure S3: Analysis of stwintron length and that of the constituting internal and external U2 introns for the two groups of [D1,2] stwintrons; Figure S4: Symmetry in ten of the uniquely occurring (older) stwintrons in *Hypoxylon* sp. CO27-5 as revealed by the alignment of each of the stwintrons with its own reverse complement sequence; Figure S5: Optimal secondary structures of nine *Hypoxylon* sp. CO27-5 stwintrons from the miscellaneous group of 81 stwintrons, identified in this work, compared to those of five *Hypoxylon* sp. CO27-5/EC38 sequence-similar sister stwintrons; Figure S6: Comparison of predicted minimum free energy (ΔG) levels for optimal secondary structure folding of sister stwintrons and the uniquely occurring (UO) stwintrons; Table S1: identifiers, localisation, statistics and other information concerning 117 sister stwintrons and uniquely occurring [D1,2] stwintrons in *Hypoxylon* sp.CO27-5 and in 19 other Hypoxylaceae; Table S2: Oligonucleotide primers used in this study; Table S3: RNA SRA reads confirming alternative splicing of [D1,2] stwintrons by one splicing reaction between the distal 5′- and 3′-splice sites, leaving the stwintron’s 5′-G_1_ exonic (frameshift +1).
