# Supplementary material for: Unique and Repeated Stwintrons (Spliceosomal Twin Introns) in the Hypoxylaceae
Source: J Fungi (Basel). 2022 Apr 13;8(4):397. doi: 10.3390/jof8040397 (PMC9024468; doi:10.3390/jof8040397)
Supplement: Supplementary file 1 [file jof-08-00397-s001.zip › Supplementary Material.pdf]

Supplementary Material (online)

associated with

Unique and repeated stwintrons (spliceosomal twin introns) in the Hypoxylaceae

Erzsébet Fekete[1]*, Fruzsina Pénzes[1,2], Norbert Ág[1], Viktória Ág-Rácz[1,2], Erzsébet Sándor[3], Claudio Scazzocchio[4,5], Michel Flipphi[1], Levente Karaffa[1,6]

[1] Department of Biochemical Engineering, Faculty of Science and Technology, University of Debrecen, H-4032 Debrecen, Hungary
[2] Juhász-Nagy Pál Doctoral School of Biology and Environmental Sciences, University of Debrecen, H-4032 Debrecen, Hungary
[3] Institute of Food Science, Faculty of Agricultural and Food Science and Environmental Management, University of Debrecen, H-4032 Debrecen, Hungary
[4] Section of Microbiology, Department of Infectious Diseases, Imperial College London, London SW7 2AZ, UK
[5] Institute for Integrative Biology of the Cell (I2BC), Université Paris-Saclay, CEA and CNRS Unité Mixte de Recherche UMR 9198, 91190 Gif-sur-Yvette, France
[6] Institute of Metagenomics, University of Debrecen, H-4032 Debrecen, Hungary

* Author for correspondence: kicsizsoka@yahoo.com

Supplementary figure S1
Supplementary figure S2
Supplementary figure S3
Supplementary figure S4
Supplementary figure S5
Supplementary figure S6

Supplementary table S2
Supplementary table S3

Supplementary table S1 is provided separately as an Excel file (Supplementary_Table_S1.xlsx): Identifiers, localisation, statistics and other information concerning 117 sister stwintrons and uniquely occurring [D1,2] stwintrons in *Hypoxylon* sp. CO27-5 and in 19 other Hypoxylaceae (2 sheets)

```
>Hypoxylon_CO27-5
CAGACACTCGATGA----g      -------atattcgatatatctcg-gagata-ctgga----ccttgttcgctaactataga-tcagtatgtaccat-tggc-cacatatgttagggtgggcttctgcc--tacgatgtaa-c---cattctgctaaccatatgacctctcttcagTTCGCTCCAACCG

>H.pulicicidum
CAGACACTTGATGA----ggttcgt-------atattcaacatatctcg-gagatc-ttaga----ctttcttagctaactgatat-ttagtatgttcttg----g-tcacaatcttatgatggactcctgtg--ctcgacttga-c---ctctctactaactgtgtacctcctc--tagTTCGCTTCAACCG

>Hypoxylon_CI-4A
GAAACTCTCGACGA----ggtttgt-------ataataagttgtccccc----gagtctgag----tattgtgtgctaactgaggc-ttagtatgttctgac-----tatttttttcaccgattactcctgac--ttta-----------gtatgttctgacaccatgattcttgctcagTTCACTTCAACCA

>Hypomontagnella.monticulosa
GAGACACTTGATGA----ggttcgt-------accgtggaagtaaacca-actact-ttcggatgccctcttttgctaactaaagc-ttagtatgccttaat-----gatggatcccagtgttcctccttgac--ttcaaagtaac----ttgttcactaacccc-----gactacatagTTCTCTCCAGCCA

>Jackrogersella.multiformis
GCAACAATCGATGA----ggttcgt-------gcgcccagtacgtatca-aacata-atatgtaggcttggtttactaatttggac-ttagtatgcacccg----a-tgagattttcagaatacttccccgat--tacaaaccag-c---ctgcccactaatcttattctacctcttcagTTCACTTCAGCCG

>Annulohypoxylon.stygium
GCAACAATTGATGA----ggtttga-------gcgcctaatatcctcca-gatcac-tgtgaactgcgtcgtttgctaacatcttt-tcagtatgcgcaatctgat-cacaatctttaagataatttcttcgg--tctcgattcgtc---ccgtctgctaattat-----ttctctctagTTCACTTCAGCCA

>Pyrenopolyporus.hunteri
GAGACGCTCGATGA----ggttcgtgccgactggggttaaatatctc---gttattctgttt----tctaatttactaactttggc-ttagtatgtattagc-----ttcggtttctggacagcctgtccagt--tttagcggtac----tgatatactgactatttaatgtgtcttcagTTCCCTTCAGCCA

>Daldinia.childiae
GAAACACTTGATGA----ggttcgt-------acccatacgtattg----aatagcctgccggttattatatttgctaactgtaaa-ttagtatgcattct------tttgtgtcctaggacaacccctggt---cttaacgga------atctctactaaccattttctctgtttctagTTCTCTGCAACCT

>Entonaema.liquescens
GAAACACTCGATGA----ggttcgt-------gacaataatatattg---aatatcttactg----tcatatttactaacgatgggcttagtatgcgttga------tctaactcctaggaaggcctaccggc--cttaatggaa-----ctctctactaactattcgctctgtccctagTTCTCTGCAACCG

>H.rubiginosum
GAAACACTCGATGA----ggttcgt-------gtggtctatacccctcc-gataacattagg----tctcgtttactgacaatatt-tcagtatgctttag----c-tcaagtttcctaaagtgtattggcat------------------ggcacactaactgtatgacttttcttagTTCGCTTCAACCC

>H.rickii
GAAACTCTGGACGAgtaaggttcgt-------gttgttgatatcccc---aatgagcccgga----tcattttgactaacaacggc-tcagtacgcataaat-----ctcagttcctagggtactcgcgaagt--cacggtgggat----attgatactaaccaaatgc-cccttcttagCTCTCTCCAACCA

>H.fragiforme
GAGACACTTGATGAgtaaggtttgt-------gttatcaacatgtccc--gataattcgaag----ctacatttactaactactgc-ttagt--------------------ttcataggatatcctcgagtt--tgacaatgaaca---gcaaaagctaaccacatgcttccgccttagCTCACTACAACCG

H.lienhwacheense
GAAACCCTTGATGAgtgaggttcgt-------attgtcaagacgtcttatattcgctttagg----tgccatttgctaaataagcc-ctagtacctaccgg---tg-accacttcctagagtatctgtccgat--cttgatataacccgttcacatattaaccaagtgctttctcattagTTCACTTCAACCG

>Xylaria.sp_JS573
GAAACGCTCGATGA----ggttagttgccaatccatctggatatt-----aatgagttctaa----ttttactgactactcagtgc-ctagtatgcaccaatagtc-cacggatttcagagtcatggg----------------------tgacctgctaacagcgt-attgtttttttagTTCCCTCCAACCG

>Xylaria.grammica
GAAACCCTTGATGA----ggtttgt-------attactctgcagccccc-ggattgttttta----tatcacatgctaacggctat-atagtacgtttcaaccctа-ggtcattaataaattggt----------tcgaattggatc---taccttgctgacgtcagcctctgataatagCTCACTTCAACCA

>Xylaria.longipes
CAAACTCTTGATGA----ggtctgt-------attaattcgcggttcct-aggtgttacttg----tataa-atgctaactgattctgtagtacggctcaacccca-tgtcggaatcaaaccgac----------ttgaactggata---tgccttgctgacatagacttctgattatagTTCACTTCAACCT

>Rosellinia.necatrix
GCGACCGTTGACGA----ggtccgt-------ataagctggttcattcacatcaatt---------tatcgcgtgctgacttcc---atagtatgcctcaatcctactattctcgccaggtcgat----------ccgaactccgta---tgctctgctaacactagcttttgactctagCTCTCTTCAACCG

>Arthrinium.malaysianum
GAAACCCTTGATGA----ggtaagccgtcacttatttcggtctatac-------------------ggtgttttgctaattgttct-ttagtatgtcatga------aaccaagcccgtgattcgtgc-------agagatgcagac---cgcctcgctaacactgg---tgcatcaaagTTCCCTGCAACCC

>Pestalotiopsis.fici
GAGACGCTGGATGA----ggtgagt-------agaggagactttgcttc-gtatgatgctgc----atgcaattgctaaccaaacttgcagtacgatgata-------ccctctccgtggtcgacttatagctatagaccttgcatt---gacttgactgacac-------gtgcttagTTCATTACAACCC
```

Supplementary Figure S1. Sequence alignment of stwintron number no-274 in orthologue genes for a monovalent cation:proton antiporter in species of *Xylariales* (*Hypoxylaceae, Xylariaceae*, and other families). In three species – *Hypoxylon rickii*, *H. lienhwacheense*, *H. fragiforme* – the phase two [D1,2] stwintron morphed into a phase two [D5,6] stwintron. The alignment was generated by MAFFT v.7 using E-INS-i iterative refinement and the 1PAM scoring matrix, with subsequent manual correction to properly align the 5' exon-stwintron junction. Intronic sequences are in lower case letter. Conserved 5'-donor, 3'-acceptor and canonical BP elements are highlighted by the magenta (internal intron) or turquoise (external intron) background. Alternating codons are underlined in the exonic sequences (capitals) bordering the stwintron. In *H. rickii* and *H. lienhwacheense*, tetranucleotides directly downstream the intron fusion point (5'-AG|T) are reminiscent of a [D1,2] split donor sequence (5'-GTACGC and 5'-GTACCT, respectively) and are highlighted in yellow.

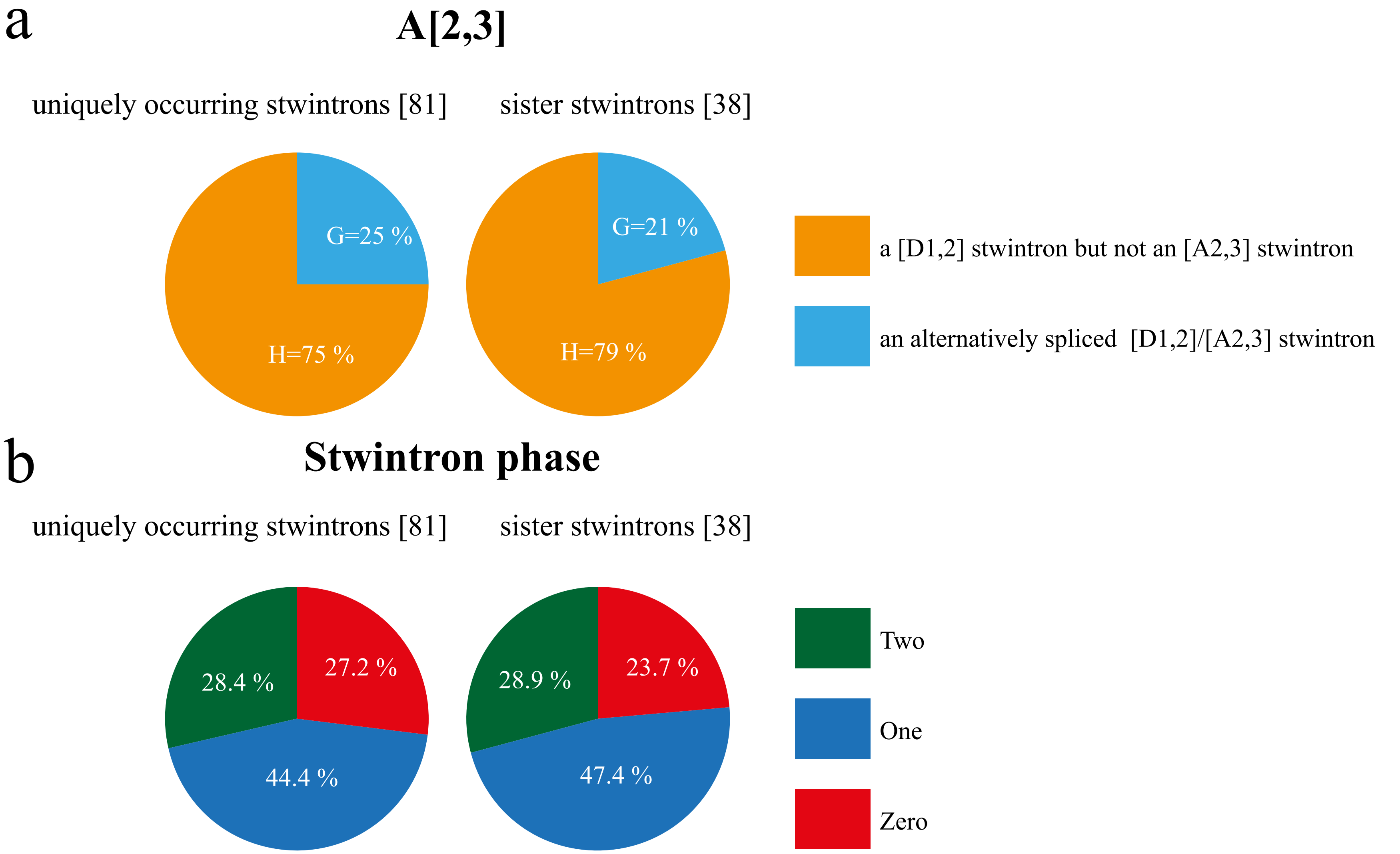

Supplementary Figure S2. Percentages of overlapping [D1,2] and [A2,3] stwintrons, and of stwintron phases. **(a)** The pie charts show the % of [A2,3] stwintrons in the miscellaneous group of the 81 UO stwintrons and that in the "control" group of the sister stwintrons (cf. [Fekete,2021]). **(b)** The pie charts show the phase distribution (in %) in the two groups of stwintrons. Most stwintrons are phase one (i.e., integrated between the first and second base of a codon) with a comparable distribution between the three phases in both groups of stwintrons.

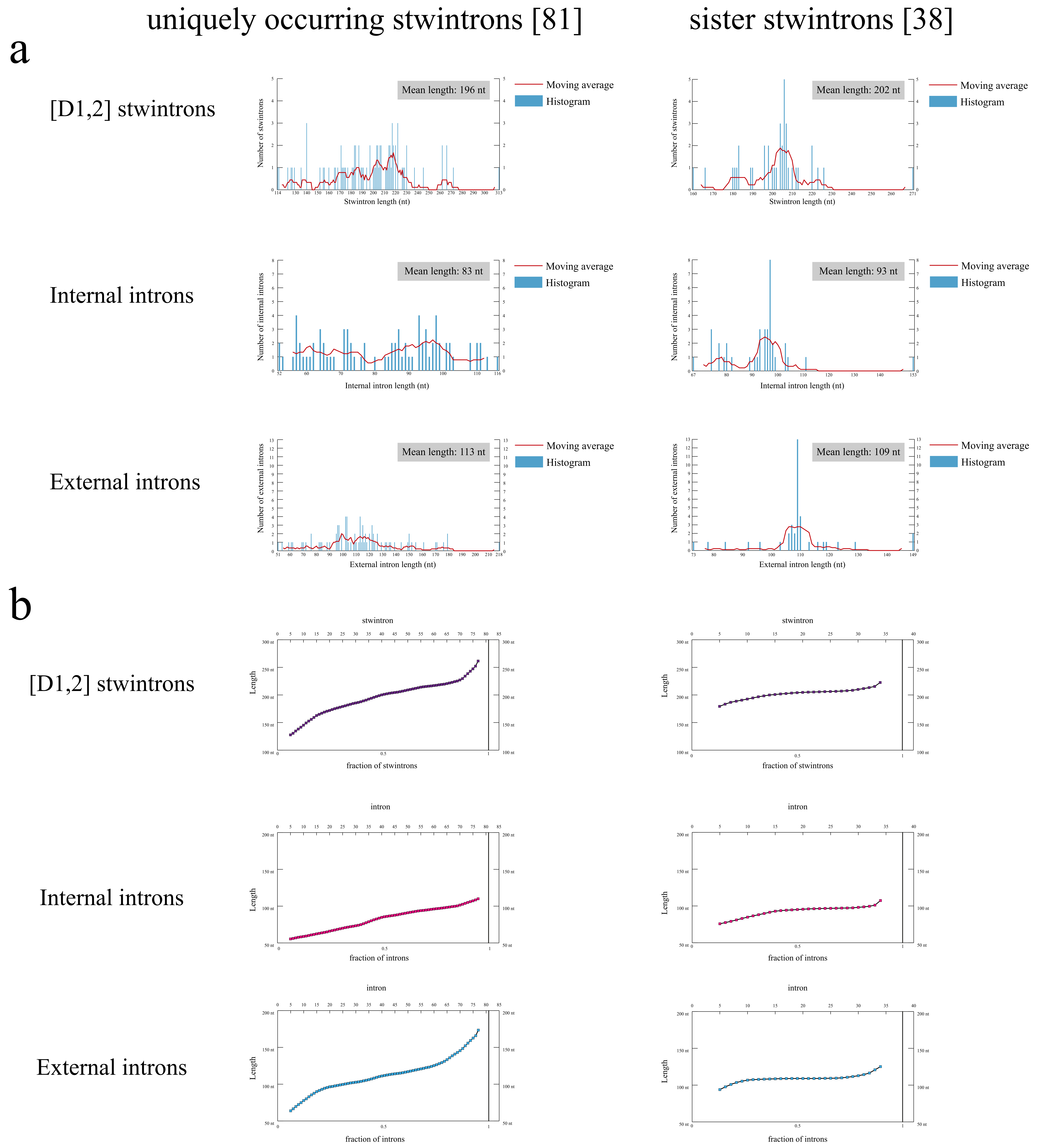

Supplementary Figure S3. Analysis of stwintron length and that of the constituting internal and external U2 introns for the two groups of [D1,2] stwintrons. **(a)** The top panels (six) show the crude data in histogram form specifying the number of stwintrons with one particular length [nt]. A moving average over a window of nine consecutive stwintrons ordered to increasing length was calculated for single step increments (red line). The mean lengths are given in the inlets at the top right of each of the six histograms. **(b)** In the bottom panels (six), incrementing moving averages of (stw)intron size (black, red and blue for the stwintrons, the internal introns and the external introns, respectively) were plotted against the fraction of stwintrons for each of the two groups.

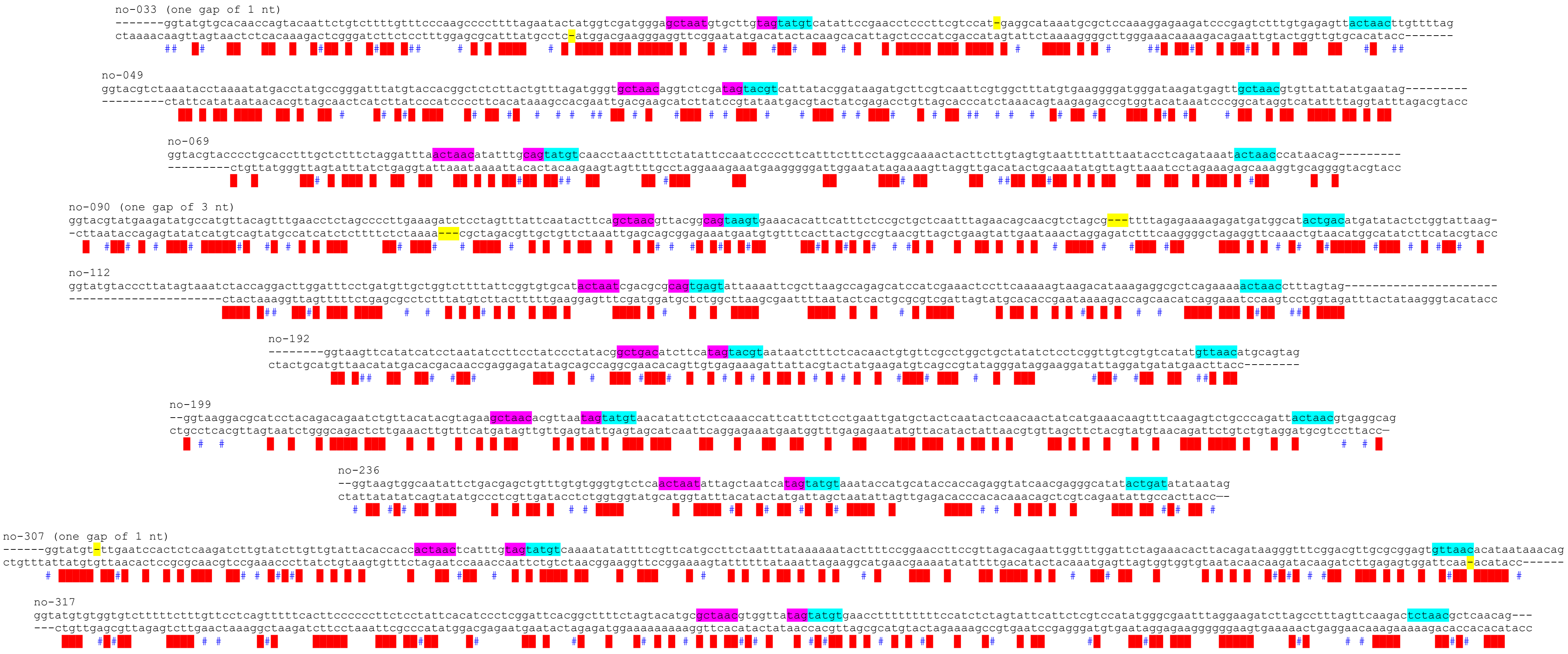

Supplementary Figure S4. Symmetry in ten of the uniquely occuring (evolutionary older) stwintrons in *Hypoxylon* sp. CO27-5 as revealed by the alignment of each of the stwintrons with its own reverse complement sequence. MAFFT alignments were carried out online, using E-INS-i iterative refinement and either of the PAM scoring matrices (200PAM; 20PAM; 1PAM). In some cases, the three scoring matrices giving the same alignment. Where multiple alignments with different symmetry midpoints were produced, alignments with the least introduced gaps and/or the shorthest gap length were preferred over those where more aligned positions were obtained as a result of more extensive gap creation and elongation by the alignment program. The ten stwintrons depicted show modest overhangs beyond the symmetrical region at either the 5' or the 3' terminus. A **red square** below the aligned sequences denotes those positions in the alignment where the nt (irrespective of its identity) in the stwintron was the same as in its reverse complement sequence. The **blue # (number) sign** denotes those locations in the alignment where noncanonical GU or UG base pairing can occur in double-stranded sections. In these situations, a G in the stwintron sequence matches an A in its reverse complement sequence at the same position in the alignment, and a T in the stwintron sequence matches a C. In all other situations, the position was left white in the graphic two-dimensional scheme below the alignment. The stwintrons were aligned at the centre of the image, to achieve a visual effect of mirroring. In the reading strand of the stwintron, the 5'-donors, the BP sequence elements and the 3'-acceptor are highlighted magenta for the internal intron and turquoise for the external intron. With the possible exception of stwintron number no-049, the local symmetry (base pairing) is not particularly pronounced at the stwintron termini. In UO stwintrons, there appear to be no equivalents of the terminal inverted repeats that typically occur in most of the 25 propagating sister stwintrons (cf. [Fekete,2021]).

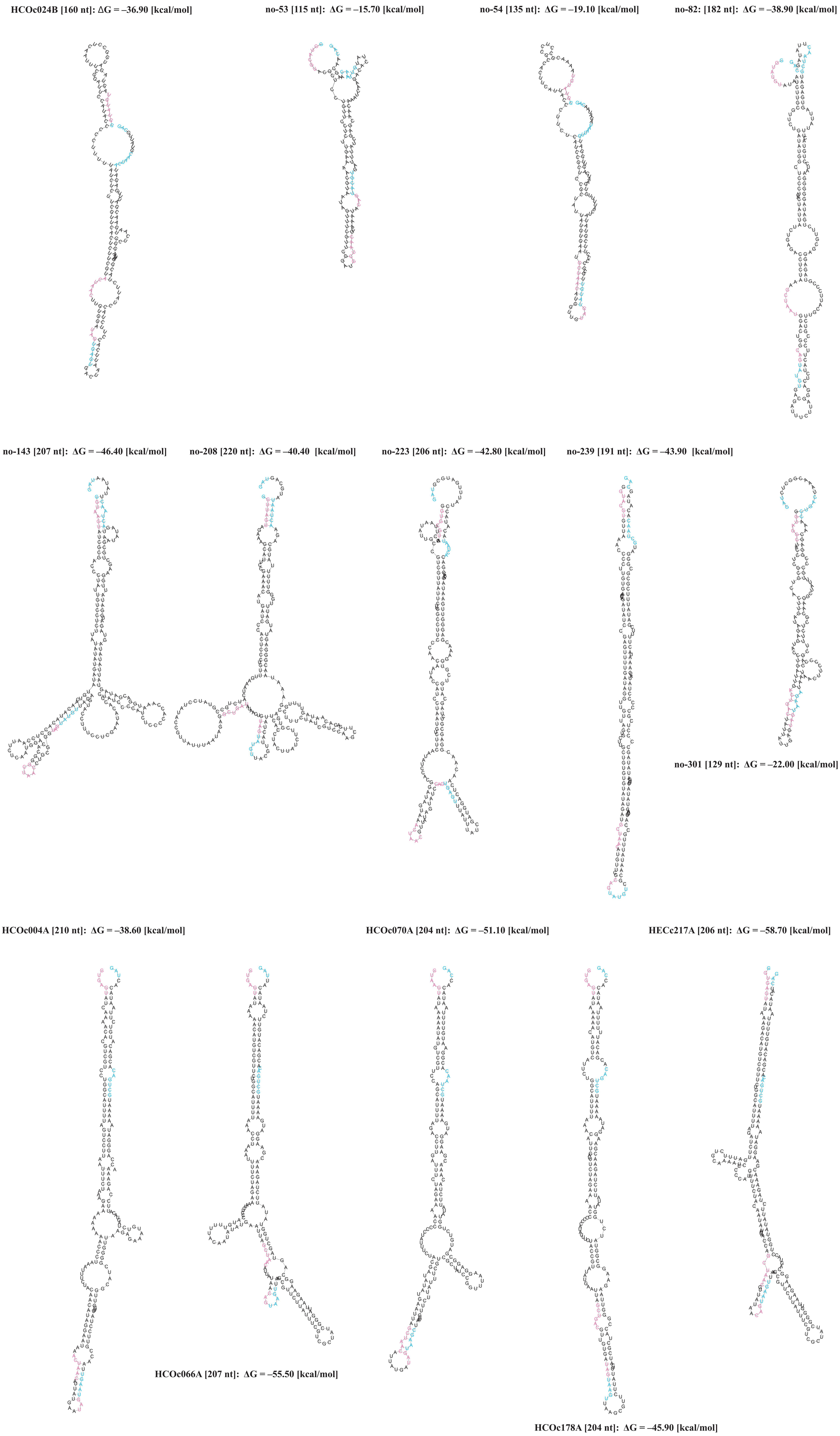

Supplementary Figure S5. Optimal secondary structures of nine *Hypoxylon* sp. CO27-5 stwintrons from the miscellaneous group of 81 stwintrons, identified in this work, compared to those of five *Hypoxylon* sp. CO27-5/EC38 sequence-similar sister stwintrons (structures at the bottom). Structures were predicted by RNAfold (default settings except that isolated base pairs were not avoided) and calculated minimal free energies (ΔG) for the proposed folding are included. 5'-Donors, BP sequence elements and 3'-acceptors were highligthed in coloured letters, magenta for the internal intron and turquoise for the external intron. Note that ΔG values correlate positively with the size of the RNA.

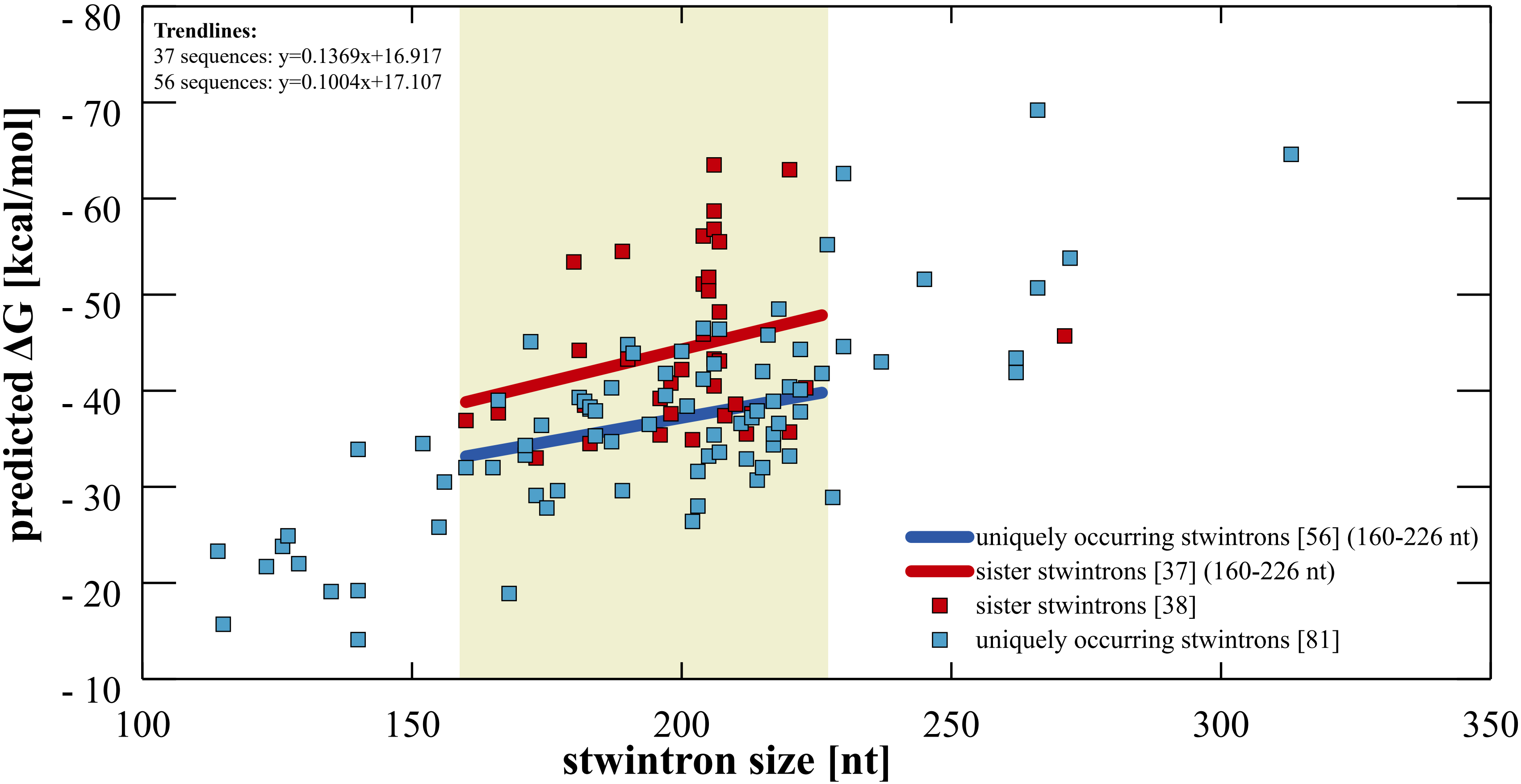

Supplementary Figure S6. Comparison of predicted minimum free energy (ΔG) levels for optimal secondary structure folding of sister stwintrons and the uniquely occurring (UO) stwintrons. The theoretical ΔG values (mimimum free energy of folding) were calculated for all 117 stwintrons with RNAfold and plotted against the stwintron length. The red squares represent the ΔG data for the 38 sister stwintrons while the blue squares are those for the 81 UO stwintrons. There is no clear distinction for ΔG values between the two groups of stwintrons. To define trend differences, we resorted to linear regression of the data within the length range between 160 nt and 226 nt, including 37 sister stwintrons and 56 UO stwintrons. The fitted trend lines (red and blue, respectively) suggest that on average, sister stwintrons have a 17–20 % lower predicted ΔG, implying that their predicted secondary structures are somewhat more stable than those of UO (evolutionary older) stwintrons of similar length. 12 sister stwintrons (~ one third) have a lower ΔG for the optimal secondary structure that any of the UO stwintrons of similar size. 18 stwintrons in the miscellaneous group of 81 UO stwintrons (also ~ one third) have higher ΔG predicted than any sister stwintron of similar size.

Supplementary Table S2. Oligonucleotide primers used in this study.

Oligonucleotide primers for **cDNA and/or gDNA** sequencing.

Gene Hypoxylon sp. CO27-5	Name of oligo	Sequence (5'-)
No 8	No8_seqF1	CGACCATTCGATATTCACGA
No 8	No8_seqR1	AATGCTAGTCGAGGCACGAG
No 8	No8_seqF2	AGATCAGTTCGCGACAATCC
No 8	No8_seqR2	AGAAACCAATGCCGACAGAC
No 37	No37_seqF1	GACCGCTGATCCTTTTCGTC
No 37	No37_seqR1	TTCCCAAATCCGAACCTCCA
No 37	No37_seqF2	CCTGAAGAGAGTGTCAATACCG
No 37	No37_seqR2	AGATAGCTCAGGGTTCGCAT
No 50	No50_seqF	GGACGCTGGACCAATTCTTC
No 50	No50_seqR	CAGCGGAGATCAGGTACCTA
No 61	No61_seqF	CGACACTTTGTGGACGGATA
No 61	No61_seqR	AGACCCAGCATCCTTAGCAA
No 69	No69_seqF1	CGAATTGGCTTCTTCGTCAC
No 69	No69_seqR1	TGCCGAAGAAATGGGGAATG
No 69	No69_seqF2	ACCCATCGTCCTTCTCTTCG
No 69	No69_seqR2	TCAAATGTACAACCACTGGCT
No 77	No77_seqF1	TTGGAACGAGGTACCAACAA
No 77	No77_seqR1	CTCGATATCCTGGGCCATAA
No 77	No77_seqF2	CGACGAGTCGTTGCTAAACA
No 77	No77_seqR2	CGTAGAAGCCTCTGGGACTG
No 82	No82_seqF	TTTTCCTGCCCGAGTTCTCA
No 82	No82_seqR	AACAGGTCTTGGCAAGTGTC
No 90	No90_seqF1	CCATGTAAGTTAATCAATCAG
No 90	No90_seqR1	TGTGTAACCGCAGATAACATA
No 90	No90_seqF2	TATGTTATCTGCGGTTACACA
No 90	No90_seqR2	CCGGCTAATAGTGCAAGGTGA
No 100	No100_seqF	AGGTAGCAGCCTGATCACGT
No 100	No100_seqR	TCTAACATGCCCGAGTCGAGC
No 132	No132_seqF1	CTCCCAATTTCGGAAAGACGT
No 132	No132_seqR1	GTACCTATGCAAAGTAATTAC
No 132	No132_seqF2	GTATGCGTTATTGGGTGGCC
No 132	No132_seqR2	ACCTTCGCAGCTCTATGGTT
No 133	No133_seqF	ATCCGAGCGATCTCGCCGATC
No 133	No133_seqR	ATAGTTTGAAGCAACTGGGAG
No 140	No140_seqF1	GTGCTGTTTTGCGAAGGTCT
No 140	No140_seqR1	CAGCAGAGCCGACACATAAG
No 140	No140_seqF2	GCCTCCCAGATTCCGAAC
No 140	No140_seqR2	AAGCAAAGATACCTCCCCGTA
No 155	No155_seqF1	AGCTCAACATCCCATCACCA
No 155	No155_seqR1	TGTGTAAGTCTGCGTCGAGA
No 155	No155_seqF2	GAGGAGGACAAGGAACCACA

No 155	No155_seqR2	CCGCACACTCCTGATAAAGC
No 177	No177_seqF	CTCTTGGTCAGCCCAGGTAA
No 177	No177_seqR	GCGTTAGTTACGATGCGAAAT
No 208	No208_seqF1	TGCAGAAGATCAGAGGTCGT
No 208	No208_seqR1	CCGTCGGTCATAGGATGTCT
No 208	No208_seqF2	ATGTCTCGGTACTTTTGCGG
No 208	No208_seqR2	TTCATGTATCGCGTTTGGGC
No 215	No215_seqF	CATAAACGCCAGATCCGCC
No 215	No215_seqR	TGGCTTCCTCAACGGTACAT
No 243	No243_seqF	AATACCTCATTCTGCTGGGC
No 243	No243_seqR	CGCTCCTTACGTTCTGTCCT
No 279	No279_seqF	CCATTCAAGCTGCCCAATCA
No 279	No279_seqR	TCCTACAGCGCTAAACCACT
No 303	No303_seqF1	CCCTACCGAACCAGATAGTGT
No 303	No303_seqR1	AGTGGCGGTGACAGAACTAT
No 303	No303_seqF2	GAGGAATTCGTTGGTGCTCC
No 303	No303_seqR2	TCATCCGTGGAAAGTAGAGCA
No 304	No304_seqF	GGCAACGAAACATAAGTGACCT
No 304	No304_seqR	AGGTGCCTCTATATGTGGGG
No 306	No306_seqF	CCCGCTTTCTAGTTCCTAATC
No 306	No306_seqR	AGAAATGCTTTTATCGCCGAC
No 307	No307_seqF1	TCGCTGAAATGCTCCCAGAT
No 307	No307_seqR1	TTAGAACCCCGTACCAGCTG
No 307	No307_seqF2	ATCGGAAATCAGGGCCATCA
No 307	No307_seqR2	TCAAAGAGAGGAGGCCGAAA
No 309	No309_seqF	TTCGCGGGCACATATCATTG
No 309	No309_seqR	AGAGGTTCAAAGGCAGACGA
No 311	No311_seqF	AACTACGGTACCCTGGTTGG
No 311	No311_seqR	TCACAGTGTTGGAATTGGCG

Oligonucleotide primers for RT-PCR verification of stwintron splicing intermediates.

Gene Hypoxylon sp. CO27-5	Name of oligo	Sequence (5'-)
No 8	No8_splinterF	TGGCGAACATCTTGGTTGCT
No 8	No8_splinterR	AATTTCGCCCCTCGAATAAT
No 37	No37_splinterF	GACCGCTGATCCTTTTCGTC
No 37	No37_splinterR	AGCAGTGAAATGAGGTAGGACA
No 50	No50_splinterF	GGCAAGAGACACGGATCAAG
No 50	No50_splinterR	AGCAAGACTAGCACAAGGAGA
No 69	No69_splinterF	ACCCATCGTCCTTCTCTTCG
No 69	No69_splinterR	GCCTAGGAAAGAAATGAAGGGG
No 71	No71_splinterF	AGACCTTGTGCTGGATCGTT
No 71	No71_splinterR	GGGTAGAAAAGATGCAACGTCA
No 77	No77_splinterF	ACTTTCGTTCGAGGGGTTTT
No 77	No77_splinterR	TTGACCACGAGAACAAGAGC
No 82	No82_splinterF	TCAAGTTGGATGCGGTGATC
No 82	No82_splinterR	TGCAAGACGGGAAGTAGAGT
No 90	No90_splinterF	TATGTTATCTGCGGTTACAC
No 90	No90_splinterR	CAGAGTATATCATGTCAGTAT
No 100	No100_splinterF	ATCGGAGTTCCCTGTTTGCT
No 100	No100_splinterR	GACCCTAAAACTTCCATGTCGG
No 115	No115_splinterF	GGAGGCCGAGCTCAAAGATA
No 115	No115_splinterR	GCCAAAGTTGAGAAGAAGGGA
No 132	No132_splinterF	TTGCGAAAGCCGTCGAGCTC
No 132	No132_splinterR	CTGGACATCAGCATTGGAGTTC
No 133	No133_splinterF	CGACTGACCAATTAAAGGCC
No 133	No133_splinterR	GTATGATTAGCAACCCTCAT
No 140	No140_splinterF	GGGGAATGCTCACCATTGTT
No 140	No140_splinterR	ATTAGCGAAGAACGCGAAAG
No 155	No155_splinterF	CGCATCGACATTCCGGAAATG
No 155	No155_splinterR	TTCGTGCCAAGATGCGTATG
No 174	No174_splinterF	TCCAACTTCTACCCCGTCAC
No 174	No174_splinterR	GCTCCTCCTTCTCTTTGGCT
No 177	No177_splinterF	CTCTTGGTCAGCCCAGGTAA
No 177	No177_splinterR	CCGAATGAAAATCGGTGGTA
No 189	No189_splinterF	AAGATTCGAGCTTGGGTCCA
No 189	No189_splinterR	CGCCTTTGATGATTTGACATGT
No 208	No208_splinterF	CTGGTGGTTATGGCTGGAGA
No 208	No208_splinterR	AACCCAAATCATACTCCCGTT
No 215	No215_splinterF	CATAAACGCCAGATCCGCC
No 215	No215_splinterR	TTCCGATTAAGCCCCTGTGT
No 243	No243_splinterF	TGAAAGCGCTCAATGATCCG
No 243	No243_splinterR	TTCGTTAGTAAGCGTCTCATGTG
No 279	No279_splinterF	TCCCAGTTCTTCTGACGGAC
No 279	No279_splinterR	GAAAGCTCGTAGTTGTCCCC

No 301	No301_splinterF	CGCCTCGGTTGTTCAGAAA
No 301	No301_splinterR	GCCGTTTAGTCAGGTTTGCT
No 302	No302_splinterF	AGGCAGCCAGGATTAATTGC
No 302	No302_splinterR	TGGGAAAGAGGAAGCGTAGA
No 303	No303_splinterF	CCCTACCGAACCAGATAGTGT
No 303	No303_splinterR	AGATCAACACTACGGGAGGA
No 306	No306_splinterF	CCCGCTTTCTAGTTCCTAATC
No 306	No306_splinterR	GAGACGCAGGTGTTTGGATC
No 311	No311_splinterF	AACTACGGTACCCTGGTTGG
No 311	No311_splinterR	TTTTCCCTTCACGGCGCA

Table S3. RNA SRA reads confirming alternative splicing of [D1,2] stwintrons by one splicing reaction between the distal 5'- and 3'-splice sites, leaving the stwintron's 5'-G_1 exonic (frameshift +1).

Stwintron number	JGI read (>jgi|HypCO275_1)	SRA read (NCBI)
HCOc004A no-3	247537|CE158765_624:240-369	SRR1801288.17896446
HCOc017A no-270	120064|CE31292_1266:794-921	
HCOc017B no-271	119130|CE30358_773:2014-2109	
HCOc047A no-249	257094|CE168322_472:629-780	
HCOc052A no-245	265540|CE176768_679:351-453	
HCOc061A no-39	279437|CE190665_61:242-336	
HCOc066A no-43	287660|CE198888_3359:1295-1517	
HCOc070A no-233	293988|CE205216_93:1-173	
HCOc102A no-218	341221|CE252449_2813:235-431	
HCOc236A no-117	187281|CE98509_2931:661-861	SRR1801290.14509435
HCOc271A no-156		SRR1801290.13818186
HCOc332A no-131	237704|CE148932_72:776-962	
HCOc378A no-134	254955|CE166183_478:339-438	SRR1801290.31839927
HCOc406A no-141	263616|CE174844_102:715-900	
HCOc522A no-144	286513|CE197741_184:551-683	
HCOc016A no-15	105281|CE16509_28:279-386	
HCOc016B no-17	105936|CE17164_560:227-377	
HCOc024B no-25	168552|CE79780_11486:1568-1690	
HCOc046A no-250	256095|CE167323_275:295-399	
HCOc091A [no-nu]	328600|CE239828_25:158-290	
HCOc159A no-90	122447|CE33675_2831:1185-1385	
HCOc304A no-129	223664|CE134892_1656:808-971	
HCOc103A [no-nu]	342627|CE253855_94:52-156	
7	332912|CE244140_201:140-296	
8	346406|CE257634_424:1164-1268	
14	105179|CE16407_251:1339-1539	
19	130513|CE41741_52:757-921	
22	150822|CE62050_1910:1382-1582	
29	214722|CE125950_1394:303-513	
33	262838|CE174066_647:698-921	
27	269919|CE181147_260:1-184	
44	288307|CE199535_2228:207-407	
48	302454|CE213682_1650:431-540	
50	309557|CE220785_1327:132-332	
54	327120|CE238348_692:280-468	
61	341505|CE252733_11:50-177	
69	351606|CE262834_13216:1833-1991	
71	357575|CE268803_515:255-358	
74	360839|CE272067_1497:1583-1654	
77	369120|CE280348_739:255-409	
90	122447|CE33675_2831:1185-1385	
91	123793|CE35021_98:374-487	

100	146315|CE57543_8577:732-887	
112	176189|CE87417_2708:375-431	
115	183209|CE94437_6:3-275	
124	206896|CE118124_21363:510-612	
125	207196|CE118424_1208:114-209	
132	237960|CE149188_2825:1076-1145	
133	241731|CE152959_667:1308-1393	
136	255988|CE167216_15939:353-414	
143	272690|CE183918_691:40-111	
155	216632|CE127860_2554:664-765	
192	133496|CE44724_4179:73-281	
199	110816|CE22044_1283:1997-2104	
200	108791|CE20019_6740:195-395	
202	101825|CE13053_135:224-335	
208	366423|CE277651_87:79-159	SRR1801289.27881864
215	352635|CE263863_47:103-252	
232	305569|CE216797_9386:1439-1496	
234	293941|CE205169_1567:625-753	
236	292136|CE203364_100:443-532	
238	289275|CE200503_1:7-78	
241	271706|CE182934_31481:1-118	
243	270072|CE181300_461:30-128	
279	247464|CE158692_1:1-101	
300	125672|CE36900_402:423-623	
301	247549|CE158777_93:564-780	
302	357547|CE268775_113:334-432	
306	155620|CE66848_2129:641-845	
307	269137|CE180365_34:263-347	
311	129693|CE40921_1533:374-470	
312	196936|CE108164_2149:253-423	
313	251091|CE162319_1704:229-343	
314	206866|CE118094_1518:309-447	
315	217032|CE128260_178:708-862	
316		SRR1801287.4140706
317	359495|CE270723_10319:506-669	
318	338896|CE250124_850:1615-1811	SRR1801292.18936684
319	138423|CE49651_4453:1220-1434	SRR1801292.16737170
320	114221|CE25449_357:632-787	
321	102312|CE13540_573:2-202	

Where multiple reads were extant, only one is given.
